# HyPIC-3D enables characterization of migratory cancer cell subpopulations in 3D hypoxic microenvironments

**DOI:** 10.1016/j.crmeth.2026.101454

**Published:** 2026-05-11

**Authors:** Luana Schito, Sergio Rey-Keim

**Affiliations:** 1 UCD School of Medicine, Dublin 4, D04 C7X2, Ireland; 2 UCD Conway Institute of Biomolecular and Biomedical Research, University College Dublin, Belfield, Dublin 4 D04 C7X2, Ireland

**Keywords:** cancer cell migration, migration assays, 3D microenvironment, tumor hypoxia, cancer spheroids, metastasis, 3D cancer models

## Abstract

Malignant tumors harbor cancer cell subpopulations with heterogeneous migratory behaviors. Identifying the molecular programs specifying whether these subpopulations migrate or remain stationary is essential for preventing metastasis and requires experimental approaches that directly separate and analyze these subpopulations as they move throughout 3D microenvironments. Here, we present “paired isolation and characterization in 3D hypoxic microenvironments” (HyPIC-3D), a method to isolate and characterize migratory and non-migratory cancer cell subpopulations from the same pool of spheroids. By adapting tissue culture inserts used for migration assays, we established a migration platform that preserves oxygen gradients and the spheroid architecture. HyPIC-3D shows that the pattern and extent of migration differ among cancer cell types while identifying distinct molecular switches and oxygen requirements underlying this heterogeneity. Integrated with image analysis tools, HyPIC-3D is implemented with standard computational and laboratory equipment and is amenable to diverse downstream applications, enabling mechanistic dissection of migration and discovery of metastatic regulators.

## Highlights

• HyPIC-3D allows collection of migrating and non-migrating cancer cells in spheroids
• HyPIC-3D enables side-by-side analysis of cancer cell migratory heterogeneity
• HyPIC-3D captures distinct markers of migration
• HyPIC-3D links intracellular hypoxia to migratory states

## Motivation

A number of technologies have been developed to investigate cancer cell migration (and invasion) in both 2D and 3D systems. However, most (1) assess motility in unpaired samples, (2) collect and analyze samples based on user-defined phenotypic features, and/or (3) require multiple processing steps between sample collection and analysis. These factors introduce inter-replicate variability and reduce statistical power to detect salient changes in motility and their correlation with molecular drivers. To address this, we developed HyPIC-3D, a method that enables direct collection and comparison of migratory and non-migratory cancer cell fractions from the same pool of 3D samples. Accordingly, HyPIC-3D uncovers intrinsic migratory heterogeneity within the original population, facilitating the identification of molecular markers driving migration. HyPIC-3D is implemented with standard tissue culture inserts and complemented by open-source image analysis tools, allowing end-users to gain mechanistic insights into cancer cell migratory heterogeneity using conventional laboratory and computational resources.

## Graphical abstract

**Figure:**
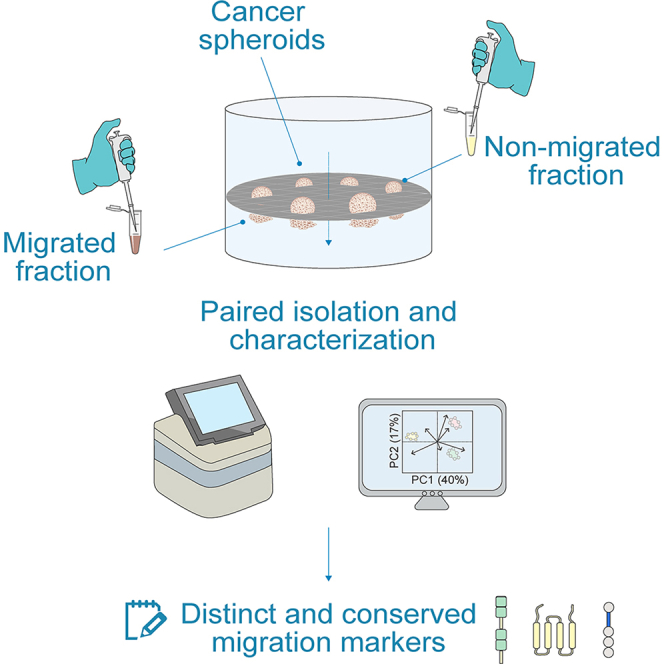

## Teaser

Schito and Rey-Keim present HyPIC-3D, a method for directly comparing migratory and non-migratory cancer cells in 3D microenvironments. This approach reveals how specific markers and oxygen levels influence cell behavior, helping to uncover mechanisms of migratory heterogeneity and identify potential targets to limit metastasis.

## Introduction

Cell migration is central to physiological and pathological processes. In cancer, migration is an early driver of disease progression by promoting metastasis whereby extracellular and intracellular signals are integrated into subcellular changes that reorganize polarity, shape, and cell-cell and cell-matrix contacts, ultimately determining whether net movement is generated.^1^^,^^2^^,^^3^ Consistently, some cancer cell subclones remain stationary, while others engage in migration, dynamically transitioning across different movement modalities, such as solitary and collective displacement.^4^^,^^5^^,^^6^ These heterogeneous behaviors are orchestrated by complex molecular programs that enable cancer cells to adapt, and move across different microenvironments encountered during dissemination, effectively dictating their metastatic potential. As a result, dissecting the molecular mechanisms underlying this migratory plasticity and heterogeneity is critical for understanding and preventing metastasis. To this end, a number of *in vitro* experimental methods have been developed to investigate migration,^7^ serving as complements for *in vivo* models of metastasis. The scratch^8^^,^^9^ and Boyden chamber^10^ assays are among the most adopted approaches, where migration-related parameters such as speed, velocity, persistence, and migratory distances (e.g., fractional/percentage, net and total) are measured in a monolayer of cancer cells moving toward a mechanically created wound or chemotactic gradient.^11^ Notwithstanding, one of the main shortcomings of these assays is that they do not typically allow the simultaneous collection of migrated and non-migrated samples, a factor that precludes the direct assessment of phenotypic heterogeneity within the same cancer cell sample/replicate. Microfluidic chips partially address this limitation by allowing collection of individually migrated and non-migrated cells at downstream outlets and inlets, respectively.^12^ However, due to low sample yield, samples are often re-cultured and expanded before molecular or functional analyses can take place; consequently, the resulting readout reflects post-culture cell phenotypes, wherein the original migratory state might change or become undetectable.^13^ Further, the development of microfluidic platforms with a higher number of channels and larger feeders has allowed to increase migratory throughput and/or sample yield for downstream, paired analyses.^13^^,^^14^^,^^15^ Nonetheless, these approaches, along with the scratch and Boyden chamber assays, examine migration in single cells, single-cell suspensions, and/or in cells constrained to 2D microenvironments that are less likely to reflect the migratory behaviors occurring when cells sense the spatial, structural, and biochemical heterogeneity of the 3D tumor microenvironment.^11^ In addition, cells constrained within planar configurations are exposed to more uniform oxygen levels, contrasting with the spontaneously generated oxygen gradients observed *in vivo.*^16^^,^^17^ This limitation renders these systems less suitable for investigating the heterogeneity of hypoxia, a well-established and ubiquitous feature of the tumor microenvironment influencing the multi-step metastatic process.^18^^,^^19^^,^^20^ As a result, phenotypes evaluated within these *in vitro* contexts might not fully reproduce the *in vivo* metastatic potential of cancer cells.

The inherent limitations of migration examined with/in 2D systems can be in part overcome by employing 3D cancer models that closely recapitulate the architecture, signaling complexity, and oxygen gradients observed *in vivo.*^21^^,^^22^^,^^23^^,^^24^ In typical 3D cancer models, spheroids, organoids, or patient-derived organoids are seeded into microfluidic chambers or cell culture (micro)plates and dishes supplemented with cell culture media or matrices, wherein their phenotypes and dynamic behaviors, such as directional cell migration, sprouting, spreading, or invasion, can be assessed by time-lapse or endpoint imaging^25^^,^^26^^,^^27^^,^^28^^,^^29^^,^^30^; similarly, markers of interest are often analyzed through *in situ* microscopy. Importantly, the workflow underlying these approaches does not typically include steps for isolation and collection of samples at the experimental endpoint. To address this, alternative methods to extract bulk samples or specific sample subpopulations for downstream molecular analysis have been developed. These include manual excision and pooling of gel-embedded organoids according to invasive phenotype,^31^ bulk spheroid collection from embedding-matrices,^28^^,^^32^ image-guided dissection of spheroid cores and their invasive edges,^26^ and selection, followed by collection, of specific photoconverted subpopulations of cells from invading spheroids.^33^^,^^34^ These methods extend the armamentarium of approaches to measure motility, while collectively posing the following challenges: (1) multiple-step processing required after the experimental endpoint and final sample collection, including 3D sample dissociation and matrix depolymerization, cell sorting, and 2D/3D cell re-culturing, which can potentially alter the migratory phenotype; (2) user-defined, imaging-based selection of core versus invasive zones, which is not devoid of operator-dependent bias; (3) dependence on specialized equipment that might not be readily available in all laboratories; and (4) disruption of *in situ* microenvironmental features such as oxygen gradients, leading to changes in hypoxic signaling pathways influencing cell motility.

To address the need for a unifying technology aimed to overcome these challenges, we developed “paired isolation and characterization in 3D hypoxic microenvironments” (HyPIC-3D), a benchtop method to isolate and characterize paired migratory and non-migratory cancer cell subpopulations directly from the same pool of cancer spheroids, wherein 3D cellular cross-talks and spontaneous microenvironmental oxygen gradients influencing migration are preserved. HyPIC-3D allows to capture migratory heterogeneity within and across cancer cell types, identify migration and hypoxia-responsive drivers underlying these behaviors, and quantify distinct oxygen requirements of each subpopulation. HyPIC-3D can be readily implemented with standard tissue culture (TC) inserts and is scalable and compatible with diverse downstream molecular applications, including high-throughput analyses. Its workflow includes open-source, built-in script pipelines for automated and semi-automated image analysis that extract morphometric and migration metrics while minimizing user bias. HyPIC-3D is designed for broad accessibility, requiring standard laboratory and computational resources, facilitating its adoption in virtually any research laboratory setting.

## Results

### HyPIC-3D workflow enables paired isolation and characterization of migratory and non-migratory cancer cell subpopulations in 3D microenvironments

**Figure 1:**
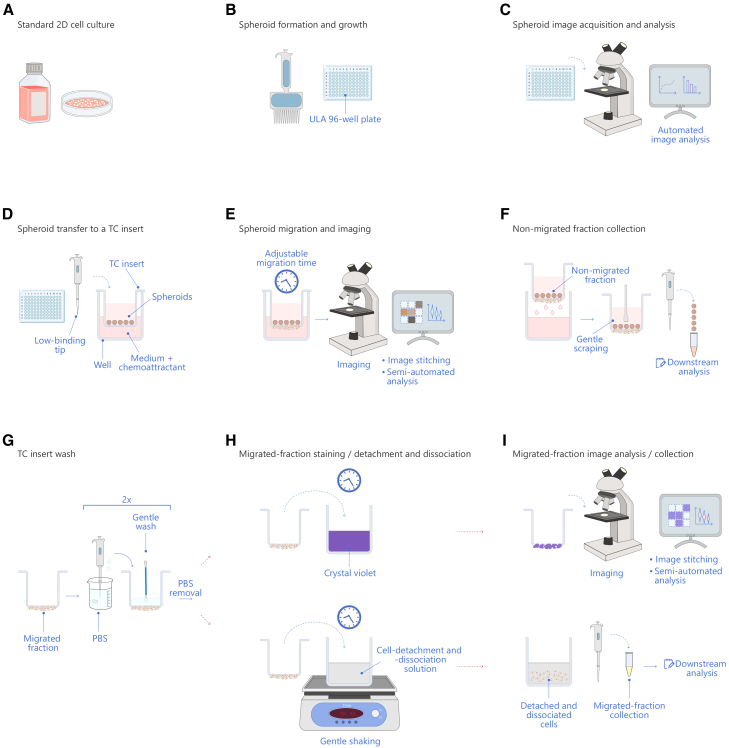
HyPIC-3D workflow for paired isolation and characterization of migrating cancer cell subpopulations in 3D hypoxic microenvironments (A) Standard 2D cancer cell culture. (B) Spheroid formation and growth in an ultra-low attachment (ULA) 96-well microplate. (C) Individual pre-migration spheroid imaging through brightfield microscopy and automated image analysis. (D) Spheroid migration assay setup. Individual spheroids are transferred from an ULA microplate to a standard TC insert and allowed to migrate through a porous PET membrane toward a 10% fetal bovine serum (FBS) gradient. (E) Spheroid migration, imaging, and semi-automated analysis at endpoint. (F) Collection of the non-migrated fraction for downstream analysis. (G) Washing of the TC insert containing the migrated fraction. (H) Migrated-fraction staining (top schematic) or detachment and dissociation (bottom schematic). (I) Imaging and semi-automated quantification of the stained migrated fraction (top schematic) and its collection for downstream analysis (bottom schematic).

The workflow of HyPIC-3D begins by culturing cancer cells under standard 2D conditions (Figure 1A). When cultures reach ≈70% confluency, cells are trypsinized, counted, resuspended as a single-cell suspension, and seeded at the desired density into a U-bottom, ultra-low attachment (ULA) 96-well microplate to induce spheroid formation (Figure 1B). After four days, the ULA microplate is removed from the TC incubator and placed under a brightfield microscope where each spheroid is imaged and morphometrically analyzed with fully automated HyPIC-3D scripts implemented in ImageJ^35^ (Figure 1C; scripts 00 and 01; Figure S1). Therefore, the pre-migration morphometric parameters, including total area, average size, fractional area, perimeter, Feret’s diameter, and circularity, can then be measured for each spheroid. Next, spheroids are gently transferred from ULA microplate wells onto a polyethylene terephthalate (PET) membrane of a standard TC insert (Figure 1D). Of note, if stacking or overlaying occurs, the TC insert can be gently tapped to spread the spheroids apart. The insert is then placed into an appropriately-sized well, and the migration chamber setup is finalized by adding medium with chemoattractant to the bottom of said well. Spheroids with their chamber are incubated under standard culture conditions, allowing migration to occur for an optimized time of 64 h (Figure 1E). This migration endpoint time can be adjusted according to end-user experimental design and hypotheses; in addition, migration dynamics can be investigated by performing time-course experiments with concomitant collection and analysis of the migrated and non-migrated fractions. Along the same line, migration chambers containing spheroids can be placed in cell culture incubators at sub-atmospheric oxygen levels (<20.9% = 21.2 kPa), effectively allowing migration under externally imposed, hypoxic conditions. It is worth to note that at post-migration endpoint, spheroids are firmly anchored to the PET membrane through a migrating sheet of cells connecting the top and bottom cell fractions, as previously observed.^36^ For post-migration analysis (64 h), the TC insert is brought onto a brightfield microscope for whole-insert imaging (Figure 1E). Multiple images covering the entire TC insert surface (i.e., "tiles") are acquired and digitally stitched into a final single output image, using the grid/collection stitching plugin (Fiji/ImageJ),^37^ eliminating the need of a slide scanner. Stitched images are next analyzed through a HyPIC-3D script designed for morphometric analysis of post-migration spheroids as laid on the top surface of the individual TC insert (Figure S2 and script 02). The TC insert is then removed from the well, and the non-migrated (top) fraction is gently scraped off and collected in a tube for downstream analysis (Figure 1F); next, the top surface is washed with PBS and gently cleaned with a sterile swab (Figure 1G). Depending on the downstream application, the TC insert containing the migrated spheroid fraction is either transferred to a well containing fixative/staining solution, such as 0.5% crystal violet in 20% methanol, for quantification of migration areas and patterns (Figure 1H, top) or transferred to a well containing a dissociating agent, such as trypsin or accutase (Figure 1H, bottom). In the former case, the stained migrated fraction is imaged with a brightfield microscope (Figure 1I, top), and multiple images covering the whole stained PET membrane are taken, digitally stitched into a single output image,^37^ and analyzed with a custom HyPIC-3D script for quantification of post-migration areas (Figure S3 and script 03). Alternatively, in the latter case, the cells from the dissociated migrated fraction are collected in a clean tube for downstream paired analysis (Figure 1I, bottom). Importantly, the HyPIC-3D pipeline can be customized to different downstream applications according to the required experimental design and sample sizes. To this end, Table 1 provides a list of parameters that can be scaled and/or modified by end-users on a per-application basis.

**Table 1:** Customization of HyPIC-3D parameters for *in vitro* metastatic 3D models

Parameter		Alternative
3D model	→	• homotypic spheroids (one cell type) • heterotypic spheroids (≥2 cell types) • patient-derived organoids • circulating tumor cell clusters • mammospheres
TC insert membrane material	→	• polycarbonate • polyethylene terephthalate (PET)
O_2_ during migration	→	• O_2_ can be set to sub- or supra-atmospheric (below or above 20.9% = 21.2 kPa) levels by using hypoxic workstations or modular incubation chambers (e.g., Billups-Rothenberg, now Embrient)
Pore diameter (μm)	→	• variable, depending on the size of migrating cell type(s). Examples: 3.0 – 5.0 – 8.0
Scraping tool	→	• mini cell scraper • micro swab
Membrane coating	→	• uncoated: migration assays • coated: invasion assays (e.g., Matrigel)
TC insert diameter	→	• variable, according to receiving culture-plate format, which scales in line with the intended downstream application
Well-plate/dish type	→	• 24-well; 12-well; 6-well
Factors determining sample yield	→	• 2D cell seeding density • 3D model growth time • number of seeded 3D spheroids/organoids • 3D migration time: affects the partition between migrated and non-migrated fractions. • chemoattractant type and concentration
Cell detachment method	→	• trypsin • accutase
Hypoxia/HIF-α reporters (for live-imaging)	→	• HRE-based fluorescent proteins^38^ • GFP-ODD and/or other fusion reporters^39^
Sample processing	→	• live cells; fixed cells; lysed cells
Image quantification	→	• provided by the included ImageJ pipelines: area, perimeter, Feret’s parameters (major/minor caliper axis length and angle), circularity, solidity, and optical density parameters (average, median, and integrated)

Non-exhaustive list of adaptable parameters within the HyPIC-3D workflow according to end-user experimental design and hypotheses. This table is meant to be illustrative rather than comprehensive. HIF-α, hypoxia-inducible factor α; HRE, hypoxia-responsive element; GFP-ODD, green fluorescent protein-oxygen-dependent degradation domain; TC, tissue culture.

### HyPIC-3D uncovers distinct and diverging 3D migratory phenotypes across cancer cell types

**Figure 2:**
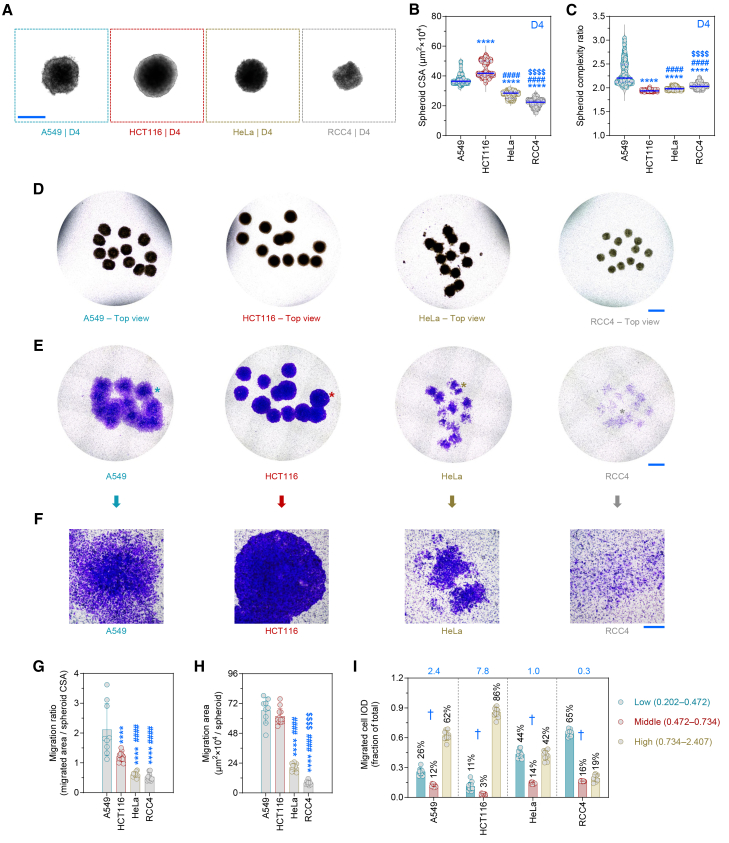
HyPIC-3D reveals distinct 3D migration patterns among four different cancer cell-of-origin types (A) Brightfield images of A549 (non-small lung), HCT116 (colorectal), HeLa (cervix), and RCC4 (renal) cancer spheroids at endpoint (day four; D4). Scale bars, 500 μm. (B and C) Automated quantification of A549, HCT116, HeLa, and RCC4 spheroid cross-sectional area (CSA; B) and complexity ratio (C) at endpoint (day four; D4). Each data point represents one image (i.e., one spheroid). Violin plots show the median (solid, blue line) and 25^th^/75^th^ quartiles (dotted, blue lines). Number of independent experiments: A549, *n* = 6; HCT116, *n* = 5; Hela, *n* = 5; RCC4, *n* = 8. ∗∗∗∗*p* < 0.0001 versus A549, ^####^*p* < 0.0001 versus HCT116, ^$$$$^*p* < 0.0001 versus HeLa by one-way ANOVA followed by Holm-Šídák multiple comparisons. Technical outliers were excluded using the Rout test (Q = 1%). (D) Stitched brightfield images of whole PET membranes showing A549, HCT116, HeLa, and RCC4 spheroids at migration endpoint (membrane viewed from above). Images were obtained by stitching four tiles. Scale bar, 850 μm. (E) Stitched crystal violet-stained images from whole PET membranes showing the migration patterns of A549, HCT116, HeLa, and RCC4 spheroids at migration endpoint. Images were obtained by stitching nine tiles. Asterisks (∗) indicate areas further magnified and shown in (F). Scale bars, 850 μm. (F) Magnified crystal violet-stained images showing A549, HCT116, HeLa, and RCC4 spheroid migration patterns. Scale bars, 250 μm. (G and H) Migration ratio (G) and migration area (H) of A549, HCT116, HeLa, and RCC4 spheroid fractions. (I) Integrated optical density (IOD) of the crystal violet^+^ signal in migrated A549, HCT116, HeLa, and RCC4 spheroid fractions; IOD is binned as low, middle, and high intensity bands and expressed as a percentage of total IOD. Each data point in (G–I) represents the average of migration ratio, area, or migrated cell IOD obtained from 12 spheroids per insert, with a total number of inserts equal to 9 (A549), 12 (HCT116), 11 (HeLa), or 9 (RCC4) across *n* = 3–5 independent experiments. ∗∗∗∗*p* < 0.0001 versus A549, ^####^*p* < 0.0001 versus HCT116, and ^$$$$^*p* < 0.0001 versus HeLa by one-way ANOVA followed by Holm-Šídák multiple comparisons. ^†^*p* < 0.05 by two-way ANOVA, followed by Holm-Šídák multiple comparisons among all four cell lines. In (G–I), data are shown as means ± SD; blue values above each cell line-of-origin in (I) indicate the high-to-low OD band ratio as a fold-change in each spheroid type.

We validated HyPIC-3D in four commonly used human cancer cell lines, representing distinct malignant tumor types, namely non-small cell lung carcinoma (A549), colon carcinoma (HCT116), cervix carcinoma (HeLa), and VHL-mutated clear renal cell carcinoma (RCC4), with the latter characterized by constitutive stabilization of hypoxia-inducible factors (HIFs),^40^ which are central mediators of the transcriptional adaptation of cancer cells to hypoxia.^19^ For each cell line, 10,000 cells/well were seeded in ULA microplates, without the addition of exogenous matrices. At day four, all cancer cell lines self-assembled into spheroids, indicating their intrinsic ability to form 3D structures in non-adherent conditions (Figure 2A). Brightfield images of each individual spheroid were then acquired and analyzed with HyPIC-3D scripts 00 and 01; accordingly, morphometric analysis of the pre-migration states revealed significant differences in the cross-sectional area (CSA) across cancer cell types, with HCT116 cells forming the largest spheroids, followed by A549, HeLa, and RCC4 cells (Figure 2B). Furthermore, we calculated the complexity ratio of each spheroid, a metric that quantifies the departure of the spheroid outline from a perfect circle and is associated with clinically agressive cancer cell phenotypes.^41^ Data showed that A549 spheroids were the least circular with the highest complexity ratio, followed by those of RCC4 and HeLa, whereas HCT116 spheroids most closely resembled a circular outline (lowest complexity ratio; Figure 2C).

At post-migration (64 h), the top surface of each TC insert was imaged as four contiguous, overlapping fields-of-view (tiles) encompassing the whole PET membrane and stitched into a single output image (Figure 2D). Post-migration imaging revealed that the non-migrated fraction of A549, HCT116 and RCC4 spheroids retained their original outline, whereas HeLa spheroids contained cell projections extending outwards from their cores, with sparse cell aggregates scattered across the membrane (Figure 2D). Of note, spheroid size was consistent within each cancer cell line, and no stacked spheroids were observed on any PET membrane. Nevertheless, when spheroids came in close contact with neighbors, some degree of post-migration fusion was observed (Figure 2D), as previously reported.^29^^,^^42^ For post-migration crystal violet staining, nine contiguous overlapping (<20% area) fields, focused on the bottom surface of the PET membrane, were acquired in order to cover the whole surface, stitched into a single output image, and analyzed. Interestingly, low-magnification images revealed that each migrated fraction was centered on an anchoring position corresponding to its non-migrated spheroid counterpart, exhibiting a distinct migratory pattern (Figure 2E). Furthermore, with the exception of RCC4 cells, migrated fractions displayed a 3D organization, containing overlapping cells rather than cell monolayers, a finding consistent with a recent report in spheroid cultures, where cancer cells migrating across membranes designed with a single hole/slit assume a 3D configuration.^36^ In addition, we examined high-magnification images of post-migration spheroids and found that A549 cells organized into cores with star-like projections radiating outwards (i.e., centrifugal spread, Figure 2F, far left), whereas HCT116 cells organized into well-rounded, dense masses without outward spread, suggesting *en bloc* migration as their main 3D migratory phenotype (i.e., centripetal spread, Figure 2F, center left). By contrast, HeLa cells migrated as irregular clusters, suggesting a highly heterogeneous migratory phenotype as they sprout from their spheroids-of-origin (Figure 2F, center right), whereas no scattered cell aggregates were observed. Interestingly, RCC4 cells migrated as single cells or small groups of cells, suggesting minimal migratory activity (Figure 2F, far right). These data collectively indicated that migration from 3D spheroids occurs preferentially through collective migratory modalities, as opposed to single-cell motility observed in planar Boyden chamber assays. Next, to quantify 3D migratory phenotypes, we developed and integrated specific algorithms into HyPIC-3D (Figures S2 and S3; scripts 02 and 03). In line with the qualitative differences seen in the crystal violet-stained images, we observed that the migration ratio, calculated as a fold-change of the migrated area versus CSA of the non-migrated spheroid fraction, ranked the four lines as A549 > HCT116 > HeLa > RCC4 (Figure 2G). Accordingly, A549 spheroids exhibited a 3.99-fold increase in the migration ratio compared to RCC4 spheroids, indicating enhanced migratory spread and speed in these lung cancer cells (Figure 2G). Of note, the experimental dynamic range for the migration ratio (i.e., ratio of the highest to the lowest value) was 9.5-fold across all spheroid types (Figure 2G). A similar hierarchy emerged for the median absolute migrated areas, where HCT116 and A549 spheroid migration areas were 61.7 × 10^4^ and 66.4 × 10^4^ μm^2^, respectively, whereas HeLa and RCC4 spheroid migration areas were significantly smaller (20.5 × 10^4^ and 8.2 × 10^4^ μm^2^, respectively; Figure 2H). To determine the degree of cellular overlap in the migrated fractions (i.e., cell stacking), used as a proxy for local migrated cell densities, we binned the integrated optical density (IOD) of the crystal violet signal into low, middle, and high OD bands (Figure 2I). This analysis showed that HCT116 migrated fractions had the highest optical density, defined as the percentage of total intensity signal above 0.734 OD units (86%), confirming their compact, *en bloc* migration. By contrast, the percentage above this threshold reached its minimum in RCC4 migrated fractions (19%), consistent with a phenotype of decreased migratory potential in these HIF-α-activated, kidney cancer-derived spheroids. Furthermore, the ratio of high-to-low OD areas in all four spheroid types (Figure 2I) resembled the ranked signal hierarchy of migration ratios (Figure 2G). Collectively, these data highlight the ability of HyPIC-3D to capture the heterogeneity of 3D cancer cell migration that is beyond the ability of conventional flat 2D assays.

### HyPIC-3D captures distinct molecular profiles in migratory and non-migratory 3D cancer cell subpopulations

**Figure 3:**
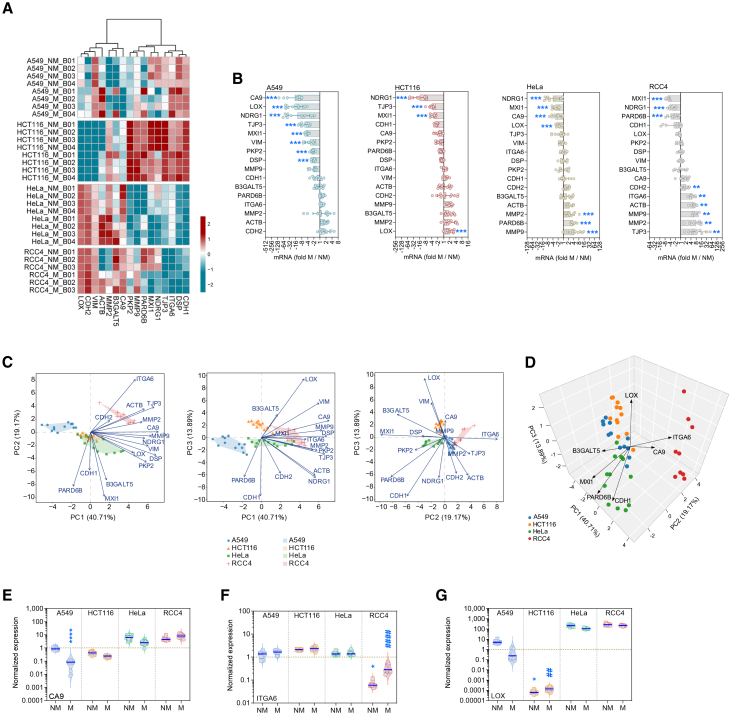
HyPIC-3D uncovers distinct molecular mediators specifying 3D cancer cell migratory and non-migratory phenotypes (A) Hierarchical clustering (Euclidean distance) and expression levels of migration- and hypoxia signaling-related gene targets in paired non-migrated (NM) and migrated (M) A549, HCT116, HeLa, and RCC4 spheroid fractions. Gene up- and downregulation are expressed as *Z* scores encoded in a color scale and calculated as the average of three technical replicates in *n* = 3–4 independent experiments (B01, B02, B03, and B04). (B) Waterfall plots showing the fold change of gene target transcript levels in paired M versus NM A549, HCT116, HeLa, and RCC4 spheroid fractions. ∗∗∗*p* < 0.001 versus NM; ∗∗*p* < 0.01 versus NM by one-sample Wilcoxon signed-rank tests. (C) PCA biplots of cancer cells (points) and gene targets (vectors) in paired M versus NM spheroid fractions. (D) 3D PCA plot of cancer cells (points) and gene targets with the highest loadings (arrows). The three major PC components are shown. (E–G) Normalized expression of *CA9* (E), *ITGA6* (F), and *LOX* (G) in paired M and NM A549, HCT116, HeLa, and RCC4 spheroid fractions. ∗*p* < 0.05 versus NM A549, ∗∗∗∗*p* < 0.0001 versus NM A549, ^##^*p* < 0.01 versus NM HCT116, and ^####^*p* < 0.0001 versus NM RCC4 by one-way ANOVA followed by Holm-Šídák multiple comparisons. Bar graphs in (B) show data expressed as means ± SD. Violin plots in (E–G) show the median (solid, blue line) and 25^th^/75^th^ quartiles (dotted, blue lines). PCA, principal component analysis; PC1, principal component 1; PC2, principal component 2; PC3, principal component 3. Number of independent experiments (*n*) = 3–4. Three technical replicates were acquired per independent experiment.

We next validated the applicability of HyPIC-3D for paired molecular analyses by performing a proof-of-concept transcriptional study comparing migrated and non-migrated 3D cell fractions, while also demonstrating the compatibility of HyPIC-3D with downstream molecular biology techniques such as reverse-transcription quantitative PCR (RT-qPCR). To this end, we extracted total RNA from both 3D fractions, synthesized cDNA, and evaluated the expression levels of a targeted panel of 16 genes mediating key aspects of cellular migration, including cytoskeletal/ECM remodeling, cell-cell adhesion, epithelial-to-mesenchymal-transition (EMT), and cell polarity; in line with the ability of HyPIC-3D to assess migration within spontaneously formed 3D hypoxic microenvironments, the 16-gene panel also included oxygen-responsive transcripts, serving as proxies for hypoxia and/or HIF-α *trans*-activation.^43^^,^^44^^,^^45^^,^^46^^,^^47^^,^^48^^,^^49^^,^^50^^,^^51^^,^^52^^,^^53^^,^^54^^,^^55^^,^^56^^,^^57^^,^^58^^,^^59^ Unsupervised hierarchical clustering of the *Z* score standardized transcript levels revealed two distinct groups, namely cluster 1 (C1), consisting of *CDH1*, *DSP*, *ITGA6*, *MMP9*, *MXI1*, *NDRG1*, *PARD6B*, *PKP2*, and *TJP3*, and cluster 2 (C2), consisting of *ACTB*, *B3GALT5*, *CA9*, *CDH2*, *LOX*, *MMP2*, and *VIM* (Figure 3A). Interestingly, C1 transcripts were higher than C2 in the highly migratory A549 and HCT116 spheroids (3D migration ratios >1.2; Figure 2G), whereas this relationship was reversed in HeLa and RCC4 spheroids, characterized by 3D migration ratios <0.75 (Figures 2G and 3A). Furthermore, targeted analysis of individual transcript changes in migrated versus non-migrated fractions (cut-off point of ≤0.5 [down] or ≥2 [up] fold and *p* < 0.01; Figure 3B) revealed that migrated A549 and HCT116 cells shared a similar transcriptional response, characterized by overall transcript downregulation, whereas HeLa and RCC4 cells presented a heterogeneous response characterized by a more balanced combination of transcript up- and downregulation (Figure 3B). Notwithstanding these differences, *NDRG1* and *MXI1* were downregulated in all migrated 3D fractions across the examined spheroid cancer types (Figure 3B), suggesting a conserved role for the MYC family of transcription factors in the migration process, while highlighting the ability of HyPIC-3D to identify conserved markers of migration.

In order to determine the potential contribution of individual or subclusters of transcripts on the observed phenotypes, we reduced the dimensionality of the RT-qPCR data by performing paired-sample principal-component analysis (PCA), analyzing the base-2 logarithm of transcript level ratios in migrated versus non-migrated cell fractions. Scree plot analysis (i.e., eigen values plotted against principal components; Figure S4) highlighted that the three top principal components, namely PC1, PC2, and PC3, accounted for ≈74% of the total transcript data variance. We next combined PC scores with individual transcript loading vectors (Table S1), using biplot analysis in order to identify individual transcripts underlying the divergent migratory behaviors observed among the four cell lines (Figures 3C and 3D; Table S2). This approach uncovered the HIF-α target *CA9* as a prominent contributor separating A549 spheroids from HCT116, HeLa, and RCC4 in PC1 (Figure 3C, left and center). By contrast, *ITGA6* separated RCC4 spheroids from A549, HCT116, and HeLa in PC1 and PC2 (Figure 3C, left and right), whereas *LOX* separated HCT116 from the migratory behaviors of A549, HeLa, and RCC4 cells in PC3 (Figure 3C, center and right). Analysis of PC1, PC2, and PC3 in a 3D scatterplot confirmed that *CA9*, *ITGA6*, and *LOX* were critical transcriptional contributors to the observed phenotypic divergence among the migrated spheroid fractions (Figure 3D and Data S1). In line with these results, geometric mean-centered transcript levels (Figures 3E–3G) revealed a 17.5-fold downregulation of *CA9* as a defining feature of migrated A549 cells (Figure 3E), prompting us to hypothesize a non-causal role of hypoxia and/or HIF-α *trans*-activity in the promotion of their migratory behaviors. By contrast, upregulation of *ITGA6* (4.7-fold in migrated/non-migrated), was a defining feature of migrated RCC4 cells (Figure 3F); notably, *ITGA6* expression was lowest in non-migrated RCC4 cells (14.5-fold below the median expression of all four cell lines), a finding potentially explaining their overall limited migration, as previously observed in a similar renal cell carcinoma cell line.^60^ Lastly, we found that *LOX* expression was nearly absent in non-migrated HCT116 cells (low limit of detection after 36 RT-qPCR cycles) and upregulated (>2-fold) in their migrated counterpart (Figure 3G). Given the role of *LOX* in promoting ECM remodeling,^61^ for example by increasing its stiffness, these results might provide a potential molecular mechanism for the *en bloc* modality of migration observed in HCT116 cells. Taken together, the findings presented herein show that HyPIC-3D enables resolving of key transcriptional differences underlying cancer cell migration within 3D hypoxic microenvironments that closely recapitulate pathophysiological malignant contexts.

### HyPIC-3D captures variation in oxygen levels between migrating and non-migrating cancer cell subpopulations

**Figure 4:**
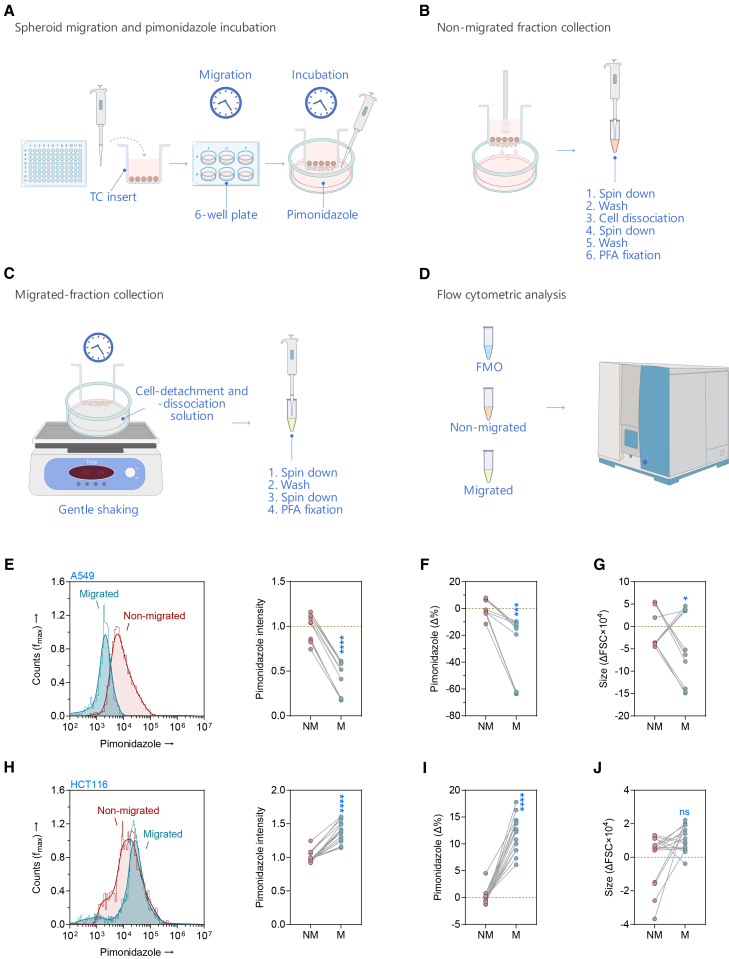
HyPIC-3D uncovers distinct oxygen profiles in migrating and non-migrating cancer cells (A–D) Four-step HyPIC-3D workflow for *in situ* pimonidazole staining and flow cytometric detection of hypoxia in paired M and NM spheroid fractions. (E) Flow cytometry histogram overlay showing an example of pimonidazole signal intensity in M and NM A549 spheroid fractions (left); paired quantification of pimonidazole signal intensity in NM versus M A549 spheroid fractions (right). (F) Percentage change of pimonidazole^+^ A549 cells in NM versus M spheroid fractions. (G) Paired analysis of flow cytometric cell size in NM versus M A549 spheroid fractions. (H) Flow cytometry histogram overlay showing an example of pimonidazole signal intensity in NM and M HCT116 spheroid fractions (left); paired quantification of pimonidazole signal intensity in NM versus M HCT116 spheroid fractions (right). (I) Percentage change of pimonidazole^+^ HCT116 cells in NM versus M spheroid fractions. (J) Paired analysis of flow cytometric cell size in NM versus M HCT116 cell fractions. FMO, fluorescence minus one; FSC, forward light-scatter; PFA, paraformaldehyde; NM, non-migrated; M, migrated. ∗*p* < 0.05, ∗∗∗*p* < 0.001, and ∗∗∗∗*p* < 0.0001 versus NM by paired Student’s *t* test; ns, not significant. Number of independent experiments (*n*) = 3. Three to four (A549) and four to eight (HCT116) technical replicates were acquired per independent experiment.

To extend the applicability of HyPIC-3D to single-cell analysis, we conducted a second proof-of-concept study measuring intracellular hypoxia by flow cytometry (Figures 4A–4D). Accordingly, we implemented the HyPIC-3D protocol up to the steps of spheroid formation and imaging (Figures 1A–1C) and then developed additional steps aimed at facilitating this analysis in both migrated and non-migrated spheroid fractions. To increase sample yield, we used TC inserts suitable for 6-well plates, while choosing to work with A549 and HCT116 cell lines endowed with high migratory activities, as determined by our previous experiments. At day four, ≈96 spheroids were transferred to a TC insert and allowed to migrate for 64 h (Figure 4A). At endpoint, a solution of pimonidazole in PBS was added to the bottom of the well, which was then gently swirled and incubated at 37°C and 5% CO_2_ for three hours (Figure 4A). Next, the 6-well plate was taken out of the incubator, and the TC insert was removed from its well (Figure 4B). The non-migrated fraction was gently scraped and collected in a tube, processed, and stored at 4°C (Figure 4B). The TC insert, now containing only the migrated fraction, was gently washed with PBS and transferred to a clean well containing trypsin, allowing cell detachment and dissociation under gentle shaking for 10 min (Figure 4C). The resulting single-cell suspension was then collected in a tube and processed similarly to its non-migrated counterpart (Figure 4C); both fractions were subsequently processed for flow cytometric detection of hypoxia (Figures 4D and S5A–S5C).

Paired analysis revealed that the migrated fraction of A549 cells had lower pimonidazole intensity and a significantly lower number of pimonidazole^+^ cells than its non-migrated counterpart, indicating lower levels of intracellular hypoxia (Figures 4E and 4F). In addition, migrated A549 cells were smaller in size (Figure 4G). By contrast, we found that the migrated fraction of HCT116 cells was hypoxic, presenting a higher number of pimonidazole^+^ cells than its non-migrated counterpart (Figures 4H and 4I), whereas no significant difference in cell size between the two fractions was detected (Figure 4J). These results suggest that differences in cell density, resulting from diverging A549 and HCT116 migration modalities, might influence the access of these cells to surrounding oxygen, thus contributing to their distinctive non-hypoxic and hypoxic migratory phenotypes. In addition, it is worth to note that variations in intrinsic oxygen consumption during migration^5^ might further contribute to the observed phenotypes. Collectively, these results demonstrate that HyPIC-3D is able to capture heterogeneous oxygen levels within distinct migratory and non-migratory cancer cell types and subpopulations, a key feature enabled by direct, paired analysis.

## Discussion

In this study we introduce HyPIC-3D, a versatile benchtop method that overcomes the challenges of current 2D and 3D migration (or invasion) systems by enabling direct recovery of migratory and non-migratory subpopulations from the same initial pool of samples at the experiment endpoint. This design eliminates subculturing steps and operator-dependent selection of samples based on qualitative migratory (or invasive) features prior to analysis. Unlike assays that assess migration in cells suspended in culture media/matrices and systems that externally impose homogenous hypoxia^62^ or rely on custom device-generated oxygen gradients,^63^^,^^64^ HyPIC-3D allows to interrogate cancer cell migration within self-organized 3D microenvironments, where phenotypic and microenvironmental heterogeneities such as native oxygen gradients are preserved. Here, we show that HyPIC-3D is able to capture a spectrum of migratory patterns, both within the same cancer cell line and across different types, ranging from radial spread or *en bloc* migration to clustered and individual modes. These findings contrast with conventional Boyden assays, where migrating cells adhere to the underside of the PET membrane as single cells or clusters, forming single or multiple layers, irrespective of the cell type, thereby preventing the detection of distinct migratory patterns across cancer cell types. Moreover, HyPIC-3D captures significant variation in migratory activity among the examined 3D spheroid cancer types. This was particularly evident in RCC4 cells, which exhibited minimal migration despite being exposed to the same assay conditions as A549, HCT116, and HeLa cells, highlighting how migration is not strictly a function of time^13^ or simply driven by passive mechanisms such as gravity^65^; rather, 3D migration appears to result from active, complex, and integrative signaling processes within the 3D microenvironment. Collectively, these results support the usefulness of HyPIC-3D as a platform for assessing migration in 3D experimental settings, closely recapitulating the dynamic context-dependent processes governing *in situ* cancer cell migration.

To validate the suitability of HyPIC-3D for paired molecular analysis, we ran proof-of-concept RT-qPCR experiments and identified key transcripts contributing to the distinct migratory behaviors observed within and across cancer cell types. We found that, in migrated A549 cells, low levels of *CA9*, a well-established and ubiquitous HIF-α transcriptional target,^45^ were a key feature underlying their spread from migratory cores, pointing to a link between reduced hypoxia and/or HIF-α *trans*-activity and this expansive (centrifugal) migratory behavior. Similarly, in migrated HCT116 cells, increased levels of *LOX* transcript, a well-known HIF-α and hypoxia-inducible target,^58^^,^^59^ suggest a role for this ECM remodeling enzyme in their *en bloc* migration modality and hypoxic phenotype. In RCC4 cells, the overall lowest levels of *ITGA6*, a marker associated with enhanced migration in kidney cancers,^60^ might account for the limited migratory activity observed in these cells. Interestingly, the analysis of our 16-transcript panel revealed consistent downregulation of *NDRG1* and *MXI1* across all migrated spheroid fractions (A549, HCT116, HeLa, and RCC4). These findings are noteworthy, as *NDRG1* and *MXI1* are functionally linked to MYC signaling,^44^^,^^66^ suggesting a conserved role for the MYC family of transcription factors in cancer cell migration. Moreover, as MYC transcriptional activity can be modulated by HIF-α through a number of O_2_-dependent and -independent mechanisms,^67^ further investigation into the identification of potentially conserved metastatic targets, by exploiting HIF-α and MYC cross-modulation within 3D microenvironments through HyPIC-3D, is warranted. It is relevant to note that the transcriptional data described herein do not constitute an exhaustive exploration; rather, they are envisioned as a proof-of-concept study that does not preclude the possibility of additional genes contributing to the specification of 3D migratory phenotypes in the examined cell lines.

It is well established that tumor hypoxia influences cell motility signaling^18^^,^^68^; therefore, determining the intracellular oxygen levels associated with migratory or stationary phenotypes is pivotal for identifying and targeting subpopulations that are more or less likely to metastasize. In line with this, HyPIC-3D allows to assess hypoxia and migratory heterogeneity in paired, 3D cancer cell populations by enabling pimonidazole staining (i.e., hypoxia detection) directly onto migratory and non-migratory fractions isolated from the same pool of samples. Accordingly, we found that the migrated and non-migrated cancer cells exhibited distinct oxygen profiles, a finding also aligning with their transcriptional responses and patterns of migration. Specifically, migrated A549 cells were less hypoxic than their non-migrated counterparts and showed reduced *CA9* expression levels and a migration pattern characterized by dispersal from central migratory cores (centrifugal), consistent with lower cell density that presumably decreases oxygen diffusion distances. By contrast, migrated HCT116 cells were more hypoxic than their non-migrated counterparts, showed increased *LOX* expression, and adopted an *en bloc* migration modality, wherein higher cell density increases the oxygen diffusion distances supporting hypoxic microenvironments. Collectively, these results suggest that the migration modality and its resulting cell density might serve as predictive factors for intracellular hypoxia when considering that oxygen diffusion is influenced by cell density at comparable oxygen consumption rates.^20^^,^^69^ In this regard, HyPIC-3D provides a framework to study how spatial organization in migration influences oxygen levels and the activation of hypoxia-inducible signaling pathways, relevant to the prediction of cancer cell responses to radio(chemo)therapy and metastasis progression.^19^

Lastly, HyPIC-3D provides automated and semi-automated image analysis pipelines for unbiased quantification of 3D cancer cell migratory phenotypes, using standard laboratory and computational equipment. In addition, HyPIC-3D includes a list of experimental parameters (Table 1) that can be adjusted to maximize sample yield while supporting a wide range of experimental design and hypothesis testing. In light of the results presented herein, we envision HyPIC-3D as a widely adoptable tool for cancer research, as the field continues to advance toward physiologically relevant and scalable 3D models closely recapitulating the *in vivo* tumor microenvironments.

### Limitations of the study

HyPIC-3D provides a flexible and accessible platform for analyzing the migratory behavior of cancer cells in spheroids; however, its applicability is inherently influenced by the ability of each cancer cell line to form cohesive 3D masses. In our proof-of-concept studies, we used cancer cell lines that self-assembled into spheroids of good compaction, a factor that allowed to transfer them from ULA microplates into TC inserts, according to the HyPIC-3D protocol. However, as previously noted,^70^ not all cancer cell lines are able to form spheroids, with many forming cell aggregates of different degrees of compaction. Therefore, strategies such as testing different methods for spheroid generation and adjusting seeding cell number and/or medium milieu^70^ need to be considered when implementing HyPIC-3D.

Another aspect to evaluate when using HyPIC-3D is the intrinsic ability of the cancer cell line under investigation to migrate in a 3D context. As observed in RCC4 cells, which exhibited minimal migration, low-migratory output can limit sample yield at experiment endpoint, potentially constraining downstream analyses such as those requiring substantial initial material. Therefore, knowledge and testing of 3D migratory abilities of the chosen cancer cell line, along with responsiveness to specific chemotactic stimuli, are key to investigate migration via HyPIC-3D.

Finally, in line with current knowledge that, within a primary tumor lesion, only a small fraction of cancer cells possess metastatic potential and, thus, effective motility,^13^^,^^71^ we observed, in both RT-qPCR and flow cytometry studies, that the migrated fraction output was consistently lower than that of its non-migrated counterpart, regardless of the cell line used. Strategies to address this aspect include testing and adjusting key experimental parameters, such as the ones provided in Table 1.

## Resource availability

### Lead contact

Requests for further information should be directed to and will be fulfilled by the lead contact, Dr. Luana Schito (luana.schito@ucd.ie).

### Materials availability

This study did not generate new unique reagents.

### Data and code availability

• Data generated via PCA are provided in Tables S1 and S2.
• The scripts for image analysis can be found at https://github.com/LS2RLab/HyPIC-3D and
• Script 00: https://doi.org/10.6084/m9.figshare.30010504
• Script 01: https://doi.org/10.6084/m9.figshare.30165115
• Script 02: https://doi.org/10.6084/m9.figshare.30165133
• Script 03: https://doi.org/10.6084/m9.figshare.30165136
• Any additional information needed to reanalyze the data reported in this paper is available from the lead contact upon request.

## Acknowledgments

We thank Associate Professor Alfonso Blanco (Flow Cytometry Core, UCD Conway Institute) for expert technical advice and Dr. Tracy Mullen (UCD Conway Institute) for laboratory management. We also thank Dr. Yoichiro Sugimoto and Professor Peter J. Ratcliffe (The Francis Crick Institute, UK) for providing RCC4 cells. Research in the authors’ laboratories is supported by the UCD Ad Astra Fellows Programme, the UCD Seed Funding Scheme from UCD, Ireland (grant nos. R20841, R20842, and SF1916 to L.S.; R20849 and R20850 to S.R.-K.), and the Eric Reid Fund for Methodology from The Biochemical Society (GB) (to L.S.).

## Author contributions

Conceptualization, L.S. and S.R.-K.; formal analysis, L.S. and S.R.-K.; investigation, L.S. and S.R.-K.; methodology, L.S. and S.R.-K.; software, L.S. and S.R.-K.; supervision, L.S. and S.R.-K.; validation, L.S. and S.R.-K.; visualization, L.S. and S.R.-K.; writing – original draft, L.S. and S.R.-K.; writing – review & editing, L.S. and S.R.-K.; funding acquisition, L.S. and S.R.-K.; project administration, L.S. and S.R.-K.

## Declaration of interests

The authors declare no competing interests.

## STAR★Methods

### Key resources table

**Table:** 

REAGENT or RESOURCE	SOURCE	IDENTIFIER
**Antibodies**		

Hypoxyprobe ™ Mab-1 antibody	Hypoxyprobe kit	Cat# HP MAb-1; RRID: AB_2801307
Goat anti-mouse IgG (H + L) antibody, Alexa Fluor 647 conjugated	Molecular Probes	Cat# A-21235; RRID: AB_2535804

**Chemicals, peptides, and recombinant proteins**		

Sodium pyruvate	Gibco	Cat# 11360070
MEM amino acids solution	Gibco	Cat# 11130051
Crystal violet	Merck	Cat# C6158-50G

**Critical commercial assays**		

MycoStrip	InvivoGen	Cat# rep-mysnc-50
RNeasy mini kit	QIAGEN	Cat# 74106
FIREScript RT cDNA synthesis mix	Solis BioDyne	Cat# 06-20-00100
Hot FIREPol EvaGreen qPCR mix plus	Solis BioDyne	Cat# 08-25-00001-10
Hypoxyprobe kit	Hypoxyprobe.com	Cat# HP1-100Kit

**Deposited data**		

Raw loadings	This paper	Table S1
Ranked loadings	This paper	Table S2

**Experimental models: Cell lines**		

A549 Human cell line	Merck	Cat# 86012804-1VL
HCT116 Human cell line	Merck	Cat# 91091005-1VL
HeLa Human cell line	Merck	Cat# 93021013-1VL
VHL-null RCC4 Human cell line	Laboratory of Dr. Peter J. Ratcliffe (Sugimoto and Ratcliffe 2022)^40^	Cell line services -The Francis Crick Institute, UK

**Oligonucleotides**		

RT-qPCR primers for 16-gene panel	This paper	Table S3

**Software and algorithms**		

CytExpert software	Beckman Coulter	RRID:SCR_017217
FlowJo v10.10.1	BD BioSciences	RRID:SCR_008520
GraphPad Prism v10.6.1	GraphPad Software	RRID:SCR_002798
Fiji v1.54p	ImageJ	RRID:SCR_002285
R Project for statistical computing v4.4.2	r-project.org	RRID:SCR_001905
Q-qPCR v1.0.4	Bio Molecular Systems	N/A
MII ImageView software v4.12	BestScope	N/A
Script 00 Automated conversion of phase contrast-images to 8-bit grayscale	This paper	https://github.com/LS2RLab/HyPIC-3D or https://doi.org/10.6084/m9.figshare.30010504
Script 01 Automated HyPIC-3D algorithm for spheroid image analysis at growth endpoint	This paper	https://github.com/LS2RLab/HyPIC-3D or https://doi.org/10.6084/m9.figshare.30165115
Script 02 Semi-automated HyPIC-3D algorithm for image analysis of spheroids at migration endpoint	This paper	https://github.com/LS2RLab/HyPIC-3D or https://doi.org/10.6084/m9.figshare.30165133
Script 03 Semi-automated HyPIC-3D algorithm for image analysis of post-migrated, crystal violet-stained spheroids	This paper	https://github.com/LS2RLab/HyPIC-3D or https://doi.org/10.6084/m9.figshare.30165136

**Other**		

Ultra-low attachment (ULA) 96-well microplate	Corning	Cat# 7007
Tissue culture inserts (24-well plates)	Sarstedt	Cat# 83.3932.800
Tissue culture inserts (6-well plates)	Sarstedt	Cat# 83.3930.800
Non-woven polyester swabs	Texwipe	Cat# TX759B

### Experimental model and study participant details

#### Cell culture

Human non-small cell lung carcinoma (A549; Cat# 86012804-1VL, Merck), colorectal adenocarcinoma (HCT116; Cat# 91091005-1VL, Merck), and cervix carcinoma (HeLa; Cat# 93021013-1VL, Merck) cells, were cultured in standard RPMI-1640 + GlutaMAX media (Cat# 61870036, Gibco), supplemented with 10% FBS (Cat# A5670701, Gibco) and 1% penicillin/streptomycin (Cat# 15140122, Gibco). VHL-null RCC4 renal cell carcinoma cells^40^ (a gift from Peter J. Ratcliffe, Francis Crick Institute, UK) were cultured in standard DMEM high-glucose media (Cat# 11965092, Gibco), supplemented with 1 mM sodium pyruvate (Cat# 11360070, Gibco) and MEM non-essential amino acids (Cat# 11130051, Gibco). All cell lines were maintained in a humidified incubator (>90% relative humidity) under non-hypoxic conditions (i.e., 19% O_2_, 5% CO_2_ and balance N_2_ at 37°C). Cell line identity was confirmed by short tandem repeat profiling (Eurofins); cell lines were routinely screened for *Mycoplasma* spp contamination (Cat# rep-mysnc-50, InvivoGen), consistently yielding negative results.

### Method details

#### Spheroid formation and growth

On the day of the experiment, ≈70% confluent cancer cell monolayers were dissociated and detached from their culture flasks with 0.05% trypsin-EDTA (Cat# 25300054, Gibco) and counted via the trypan blue exclusion assay in an automated cell counter (Countess 3, Thermo Fisher: viability >95%). Single-cell suspensions (10^5^ cells/mL) were transferred to a sterile basin. Next, 100 μL aliquots (10^4^ cells) were seeded into a 96-well round-bottom, ULA microplate (Cat# 7007, Corning), prefilled with 100 μL of culture medium with a 12-channel pipette, resulting in 10^4^ cells resuspended in 200 μL media/well. ULA microplates were then spun down (500 × g, 5 min, no brake) and incubated undisturbed for four days under non-hypoxic conditions.

#### Migration assays and sample collection

Cancer spheroids grown for four days were gently transferred with their own spent conditioned medium to a TC insert (24-well or 6-well size; Cat# 83.3932.800 or Cat# 83.3930.800, Sarstedt) and allowed to settle by gravity, a process occurring within seconds. Then, the medium volume within the TC insert was adjusted to ≈80–100 μL (24-well size) or ≈1 mL (6-well size), ensuring not to touch or move the spheroids with the pipette tip. The TC insert was carefully placed into its respective well subsequently filled with ≈700 μL (24-well size) or ≈3 mL (6-well size) of culture medium supplemented with 10% FBS (chemoattractant type and concentration can be optimized as needed), thereby finalizing the migration chamber setup. The chamber was then incubated at 37°C, 19% O_2_, and 5% CO_2_ for 64 h. At migration endpoint, the chamber was brought to a lab bench for sample collection; accordingly, the non-migrated spheroid fraction (top of the PET membrane) was gently detached with a non-woven swab and transferred into a 15 mL conical tube using a 1000-μL pipette. The PET membrane was then washed twice with ≈200 μL (24-well size) or ≈1 mL (6-well size) of room temperature PBS and this volume collected into the same 15 mL conical tube; this step can be repeated as necessary to ensure that the whole non-migrated spheroid fraction is collected. Two additional washes were then performed with cotton swabs, while discarding spent PBS. The TC insert, now containing only the migrated fraction (bottom of the PET membrane), was then transferred to a 24-well or 6-well plate containing cell detachment solution (e.g., trypsin) and gently shaken for ≈10 min. The resulting single-cell suspension was then collected into a clean 15 mL tube. Both non-migrated and migrated fractions were spun down and processed for RNA extraction.

#### Crystal violet staining

Staining of the migrated fraction for morphometric analysis was carried out only in TC inserts of 24-well format. At migration endpoint, inserts within their wells were imaged on a phase contrast microscope (see details in ‘Microscopy and digital image acquisition’) and then placed on a lab bench. The non-migrated spheroid fraction was gently scraped off and inserts washed as described above. TC inserts, now containing only the migrated cell fraction, were transferred to a well of a 24-well plate pre-filled with ≈700 μL of a 0.5% (w/v) solution of crystal violet in 20% methanol and stained for 10 min at room temperature. Inserts were then washed with PBS, dried ≥48 h in the dark, imaged, and stored in clean boxes.

#### Microscopy and digital image acquisition

Imaging of ULA microplates was performed at room temperature on a phase-contrast microscope equipped with a 25-MP CMOS camera (Sony IMX533). Images were captured using Plan Achromat light-transmission objectives (×2: 2.23 μm/px, NA 0.06; ×4: 1.11 μm/px, NA 0.13; ×10: 0.45 μm/px, NA 0.25 [Olympus]) at a resolution of 2464 × 2464 px under fixed LED illumination and at a fixed 10-ms exposure using the MII ImageView software (v4.12). Flat-field correction was applied before image capture. When the field-of-view was >22 mm in diameter, images were automatically stitched using the Fiji/ImageJ suite (v1.54p).^37^ Individual spheroid images were converted to 8-bit grayscale using a custom Fiji script allowing unattended processing (Figure 2A and script 00: https://doi.org/10.6084/m9.figshare.30010504).

#### Image analysis and quantification

In order to ensure consistent, unbiased processing and quantification of morphometric and migration parameters, we coded four customized scripts using ImageJ JavaScript. These scripts are designed to be used sequentially within the HyPIC-3D workflow and can be readily loaded into Fiji/ImageJ (Menu: Plugins→Macros→Install). All scripts require input files in a single input folder. Output text files and images are located in an automatically-created folder after each run. Images from spheroids previously photographed *in situ* within ULA microplates, were analyzed using a semi-interactive approach where the border effects observed in U-shaped wells were eliminated (Figure S1 and script 01: https://doi.org/10.6084/m9.figshare.30165115; Figure S1). For post-migration assays, the average cross-sectional area of the migrating spheroids (Figure S2 and script 02: https://doi.org/10.6084/m9.figshare.30165133), and total invasion areas (Figure S3 and script 03: https://doi.org/10.6084/m9.figshare.30165136) were measured by interactively outlining the PET membrane border, effectively eliminating TC insert walls that would otherwise impede accurate quantification. The output from scripts 02 and 03 was scaled and calibrated to determine cross-sectional areas of the non-migrated and migrated fractions (μm^2^), as well as migration ratios, used as metrics to establish the degree of centrifugal migration from spheroids derived from different cancer cell types. In addition, migration pixel intensity values were converted onto optical density by using the expression OD = log [255/*I*], where OD is the optical density of an individual pixel and I is the pixel intensity in an 8-bit image (range 0–255). Integrated OD was calculated as the sum of the OD values for all individual pixels (script 03). Since intensities of zero correspond to saturated pixels and are mathematically undefined by the OD calibration formula, all measurements of pixel intensities were constrained between 254 (OD = 0.002) and 1 (OD = 2.407). Quantitative analysis of blank TC inserts showed that a fixed threshold (OD>0.2) was sufficient to exclude background noise caused by pores in the PET membrane under fixed illumination and image acquisition. OD intensity analysis bands were therefore set as: low (0.202<OD<0.472), middle (0.472<OD<0.734) and high (0.734<OD<2.407); these bands are equivalent to percentiles 0–50 (low), 50–70 (middle) and 70–100 (high) expressed as 8-bit pixel values, enabling the analysis OD signal intensity distributions to estimate cellular density in TC inserts stained with crystal violet.

The degree of compaction and the geometry of spheroids before migration were quantified using the complexity ratio (*R*_*c*_), defined as:

$$R_{c}=\frac{P}{2\pi \sqrt{\frac{A}{\pi }}}$$

Where *P* is the perimeter (μm) and *A* is the cross-sectional area (μm^2^) of individual spheroids (calculated by script 01). In this context, a complexity ratio of one indicates a perfect circle, whereas values above one indicate an irregular border, wherein spheroid geometry deviates from a perfect circle.^41^

In order to quantify the extent and pattern of migration in cancer cells originating from spheroids, we devised a simple metric referred to as the ‘migration ratio’ (*R*_*m*_), defined as:

$$R_{m}=\frac{A_{m}}{S_{CSA}\times n}$$

Where *R*_*m*_ is the migration ratio, *A*_*m*_ is the migration area (μm^2^ from script 03), *S*_*CSA*_ is the cross sectional area (CSA) of all spheroids at the end of the migration assay (μm^2^; from script 02) and *n* is the number of spheroids laid on the insert (from script 02). A migration ratio of zero indicates no migration, whereas high ratios (>1) suggest enhanced ‘centrifugal’ migration, whereas low ratios (<1) suggest a ‘centripetal’ pattern, where migrating cells replicate the outline of their spheroids-of-origin. We posit that integrating migration ratios with OD intensity measurements can provide quantitative data on the degree and pattern of migration of cancer cells through the TC insert as they migrate away from the spheroid-of-origin.

#### Spheroid number optimization

At day 4, spheroids were transferred onto TC inserts suitable for 24- or 6-well plates using a cut 1000-μL tip. The number of spheroids and the corresponding TC insert format used in migration assays were optimized according to downstream experimental analysis and the ability of individual cancer cell lines to migrate in a 3D configuration (Figure 2). For morphometric image analysis, 12 spheroids per 24-well TC insert were used in all cell lines. For RNA extraction and gene expression analysis, a total of 48 spheroids seeded either across four 24-well TC inserts (i.e., 12 spheroids per insert) or in a single 6-well TC insert, were used in A549, HCT116 and HeLa cells; for RCC4 cells, between ≈192 and ≈240 spheroids were seeded either in a single 6-well TC insert or split across two 6-well TC inserts. For flow cytometric analysis, a total of ≈96 spheroids seeded in a single 6-well TC insert were used. This range of spheroid numbers was used in each independent experiment. As shown in Table 1, spheroid numbers can be adjusted according to experimental requirements.

#### RT-qPCR and transcriptional analysis

Total RNA was extracted from migrated and non-migrated cell fractions with a column-based RNA isolation method (RNeasy Mini Kit, Cat# 74106, QIAGEN). RNA integrity and the absence of protein contamination were spectrophotometrically confirmed (Nanodrop 2000). Reverse transcription was achieved with an MMV-based mix containing oligo-dT and random primers (typical RNA input per reaction: 0.5 to 2 μg; FIREScript RT, Cat# 06-20-00100, Solis BioDyne). Quantitative real-time PCR was performed using Hot FIREPol EvaGreen qPCR Mix Plus (Cat# 08-25-00001-10, Solis BioDyne) while transcript levels were estimated by an efficiency-corrected relative-expression method.^72^^,^^73^ Target transcripts were adjusted for total mRNA variation by determining the geometric mean of the three most stable reference genes among a panel of reference transcripts (i.e., TBP, RPL13A and YWHAZ) determined by a variance-estimation approach.^74^ To generate RT-qPCR array heatmaps, normalized expression values were log2-transformed, standardized to Z-scores, color coded and clustered using Euclidean distances to assess similarities in transcriptional expression patterns. Individual transcript expression was calculated as the ratio of normalized expression values between migrated and non-migrated cell fractions using a biological significance threshold of 0.25 < fold-change < 4 combined with a statistical significance threshold of *α* < 0.01. Principal component analysis was carried out using the *prcomp* package and *ggplot* packages within R (v4.4.2). The threshold for dimensionality reduction was determined using scree plot. Static biplots and an interactive three-dimensional principal component scatterplot were generated to determine principal component scattering and individual transcript loadings.

#### Flow cytometric quantification of hypoxia

On the day of the experiment, individual migration chambers (TC inserts of 6-well size) were moved to a tissue-culture hood, wherein 200 μM pimonidazole (Cat# HP1-100Kit, Hypoxyprobe kit) in PBS was directly added to the side of the bottom well. The chamber was gently swirled and incubated at 37°C and 5% CO_2_ for three hours. After incubation, the insert was removed; both non-migrated and migrated spheroid fractions were collected, trypsinized, pelleted, washed once in PBS, and fixed in 2% paraformaldehyde/PBS for two days at 4°C. Fixed single-cell suspensions were spun down (500 × g, 5 min), washed with 1000 μL of 1% BSA/PBS and incubated overnight at 4°C under gentle rotation in staining solution consisting of anti-pimonidazole antibody (1:250; Cat# HP MAb-1, Hypoxyprobe kit) in PBS, 1% BSA and 0.05% Triton X-100. An aliquot of each fraction was processed without primary antibody to serve as the fluorescence-minus-one control. The next day, samples were pelleted, washed in PBS, resuspended in 1000 μL of 1% BSA/PBS, and incubated with an Alexa Fluor 647 conjugated, goat anti-mouse (1:1000; Cat# A-21235, Molecular Probes) secondary antibody for one hour at room temperature in the dark under gentle rotation. After a final wash in 1% BSA/PBS, samples were analyzed on a CytoFLEX-S flow cytometer (≥10^4^ total events per biological replicate). The cytometric gating strategy is illustrated in Figure S5.

### Quantification and statistical analysis

#### Statistical analysis

The number of independent experiments and technical replicates are stated within each figure legend. Fold-change data were logarithm-transformed prior to parametric statistical analyses. Statistical differences between two experimental conditions (i.e., NM versus M) were determined by paired Student t-tests or Wilcoxon signed-rank tests, whereas comparisons among three or more experimental groups were carried out by one- or two-way ANOVA followed by appropriate *post hoc* comparisons, as stated in each figure legend. Nonlinear fitting of experimental data was assessed by F-tests. All statistical tests are two-sided and use a threshold of α ≤ 0.05 unless noted otherwise.

## Supplemental information

Document S1. Figures S1–S5 and Tables S1–S3

Data S1. Interactive PCA HTML plot of migrated versus non-migrated cancer cell transcripts, related to Figure 3

Document S2. Article plus supplemental information

## References

[1] Alonso-Matilla R., Provenzano P.P., Odde D.J. (2025). Physical principles and mechanisms of cell migration. NPJ Biol. Phys. Mech..

[2] Stuelten C.H., Parent C.A., Montell D.J. (2018). Cell motility in cancer invasion and metastasis: insights from simple model organisms. Nat. Rev. Cancer.

[3] Wu J.-S., Jiang J., Chen B.-J., Wang K., Tang Y.-L., Liang X.-H. (2021). Plasticity of cancer cell invasion: Patterns and mechanisms. Transl. Oncol..

[4] Merino-Casallo F., Gomez-Benito M.J., Hervas-Raluy S., Garcia-Aznar J.M. (2022). Unravelling cell migration: defining movement from the cell surface. Cell Adh. Migr..

[5] Parlani M., Jorgez C., Friedl P. (2023). Plasticity of cancer invasion and energy metabolism. Trends Cell Biol..

[6] Yamada K.M., Sixt M. (2019). Mechanisms of 3D cell migration. Nat. Rev. Mol. Cell Biol..

[7] Pafitanis S., Zacharia L.C., Stylianou A., Gkretsi V. (2025). In vitro models: Can they unravel the complexities of cancer cell metastasis?. Biochim. Biophys. Acta. Rev. Cancer.

[8] Liang C.-C., Park A.Y., Guan J.-L. (2007). In vitro scratch assay: a convenient and inexpensive method for analysis of cell migration in vitro. Nat. Protoc..

[9] Castor L.N. (1968). Contact regulation of cell division in an epithelial-like cell line. J. Cell. Physiol..

[10] Boyden S. (1962). The chemotactic effect of mixtures of antibody and antigen on polymorphonuclear leucocytes. J. Exp. Med..

[11] Galarza S., Kim H., Atay N., Peyton S.R., Munson J.M. (2020). 2D or 3D? How cell motility measurements are conserved across dimensions in vitro and translate in vivo. Bioeng. Transl. Med..

[12] Chen Y.-C., Allen S.G., Ingram P.N., Buckanovich R., Merajver S.D., Yoon E. (2015). Single-cell Migration Chip for Chemotaxis-based Microfluidic Selection of Heterogeneous Cell Populations. Sci. Rep..

[13] Yankaskas C.L., Thompson K.N., Paul C.D., Vitolo M.I., Mistriotis P., Mahendra A., Bajpai V.K., Shea D.J., Manto K.M., Chai A.C. (2019). A microfluidic assay for the quantification of the metastatic propensity of breast cancer specimens. Nat. Biomed. Eng..

[14] Chen Y.-C., Humphries B., Brien R., Gibbons A.E., Chen Y.-T., Qyli T., Haley H.R., Pirone M.E., Chiang B., Xiao A. (2018). Functional Isolation of Tumor-Initiating Cells using Microfluidic-Based Migration Identifies Phosphatidylserine Decarboxylase as a Key Regulator. Sci. Rep..

[15] Chen Y.-C., Sahoo S., Brien R., Jung S., Humphries B., Lee W., Cheng Y.-H., Zhang Z., Luker K.E., Wicha M.S. (2019). Single-cell RNA-sequencing of migratory breast cancer cells: discovering genes associated with cancer metastasis. Analyst.

[16] Pettersen E.O., Larsen L.H., Ramsing N.B., Ebbesen P. (2005). Pericellular oxygen depletion during ordinary tissue culturing, measured with oxygen microsensors. Cell Prolif..

[17] Grimes D.R., Kelly C., Bloch K., Partridge M. (2014). A method for estimating the oxygen consumption rate in multicellular tumour spheroids. J. R. Soc. Interface.

[18] Schito L., Rey-Keim S. (2023). Hypoxia signaling and metastatic progression. Semin. Cancer Biol..

[19] Schito L., Rey-Keim S. (2025). Editorial - Hypoxia as a molecular driver of cancer progression. Semin. Cancer Biol..

[20] Rey-Keim S., Schito L. (2024). Origins and molecular effects of hypoxia in cancer. Semin. Cancer Biol..

[21] Nath S., Devi G.R. (2016). Three-dimensional culture systems in cancer research: Focus on tumor spheroid model. Pharmacol. Ther..

[22] Crouigneau R., Li Y.-F., Auxillos J., Goncalves-Alves E., Marie R., Sandelin A., Pedersen S.F. (2024). Mimicking and analyzing the tumor microenvironment. Cell Rep. Methods.

[23] Tevlek A., Kecili S., Ozcelik O.S., Kulah H., Tekin H.C. (2023). Spheroid Engineering in Microfluidic Devices. ACS Omega.

[24] Drost J., Clevers H. (2018). Organoids in cancer research. Nat. Rev. Cancer.

[25] Suh Y.J., Pandey M., Segall J.E., Wu M. (2022). Tumor spheroid invasion in epidermal growth factor gradients revealed by a 3D microfluidic device. Phys. Biol..

[26] Weiss F., Atlasy N., van Reijmersdal V., Stunnenberg H., Hulsbergen-Veelken C., Friedl P. (2022). 3D spheroid culture to examine adaptive therapy response in invading tumor cells. In Vitro Model.

[27] Vinci M., Box C., Zimmermann M., Eccles S.A. (2013). Tumor spheroid-based migration assays for evaluation of therapeutic agents. Methods Mol. Biol..

[28] Ling Yu L.F. (2025). The impact of 3D tumor spheroid maturity on cell migration and invasion dynamics. Biochem. Eng. J..

[29] Ning K., Xie Y., Sun W., Feng L., Fang C., Pan R., Li Y., Yu L. (2025). Non-destructive in situ monitoring of structural changes of 3D tumor spheroids during the formation, migration, and fusion process. eLife.

[30] Munson J.M., Bellamkonda R.V., Swartz M.A. (2013). Interstitial flow in a 3D microenvironment increases glioma invasion by a CXCR4-dependent mechanism. Cancer Res..

[31] Henriet E., Knutsdottir H., Grasset E.M., Dunworth M., Haynes M., Bader J.S., Ewald A.J. (2023). Triple negative breast tumors contain heterogeneous cancer cells expressing distinct KRAS-dependent collective and disseminative invasion programs. Oncogene.

[32] Lehmann S., Te Boekhorst V., Odenthal J., Bianchi R., van Helvert S., Ikenberg K., Ilina O., Stoma S., Xandry J., Jiang L. (2017). Hypoxia Induces a HIF-1-Dependent Transition from Collective-to-Amoeboid Dissemination in Epithelial Cancer Cells. Curr. Biol..

[33] Khatib T.O., Amanso A.M., Knippler C.M., Pedro B., Summerbell E.R., Zohbi N.M., Konen J.M., Mouw J.K., Marcus A.I. (2023). A live-cell platform to isolate phenotypically defined subpopulations for spatial multi-omic profiling. PLoS One.

[34] Yoon S.B., Chen L., Robinson I.E., Khatib T.O., Arthur R.A., Claussen H., Zohbi N.M., Wu H., Mouw J.K., Marcus A.I. (2024). Subpopulation commensalism promotes Rac1-dependent invasion of single cells via laminin-332. J. Cell Biol..

[35] Schindelin J., Arganda-Carreras I., Frise E., Kaynig V., Longair M., Pietzsch T., Preibisch S., Rueden C., Saalfeld S., Schmid B. (2012). Fiji: an open-source platform for biological-image analysis. Nat. Methods.

[36] Kaneda S., Kawada J., Shinohara M., Kumemura M., Ueno R., Kawamoto T., Suzuki K., Kim B., Ikeuchi Y., Sakai Y. (2019). Boyden chamber-based compartmentalized tumor spheroid culture system to implement localized anticancer drug treatment. Biomicrofluidics.

[37] Preibisch S., Saalfeld S., Tomancak P. (2009). Globally optimal stitching of tiled 3D microscopic image acquisitions. Bioinformatics.

[38] Godet I., Shin Y.J., Ju J.A., Ye I.C., Wang G., Gilkes D.M. (2019). Fate-mapping post-hypoxic tumor cells reveals a ROS-resistant phenotype that promotes metastasis. Nat. Commun..

[39] Marchetti M., Ronda L., Cozzi M., Bettati S., Bruno S. (2023). Genetically Encoded Biosensors for the Fluorescence Detection of O2 and Reactive O2 Species. Sensors (Basel).

[40] Sugimoto Y., Ratcliffe P.J. (2022). Isoform-resolved mRNA profiling of ribosome load defines interplay of HIF and mTOR dysregulation in kidney cancer. Nat. Struct. Mol. Biol..

[41] Brouwer N.P.M., Khan A., Bokhorst J.-M., Ayatollahi F., Hay J., Ciompi F., Simmer F., Hugen N., de Wilt J.H.W., Berger M.D. (2024). The Complexity of Shapes: How the Circularity of Tumor Nodules Affects Prognosis in Colorectal Cancer. Mod. Pathol..

[42] Pan R., Lin C., Yang X., Xie Y., Gao L., Yu L. (2024). The influence of spheroid maturity on fusion dynamics and micro-tissue assembly in 3D tumor models. Biofabrication.

[43] Zhou Q., Dai J., Chen T., Dada L.A., Zhang X., Zhang W., DeCamp M.M., Winn R.A., Sznajder J.I., Zhou G. (2017). Downregulation of PKCζ/Pard3/Pard6b is responsible for lung adenocarcinoma cell EMT and invasion. Cell. Signal..

[44] Corn P.G., Ricci M.S., Scata K.A., Arsham A.M., Simon M.C., Dicker D.T., El-Deiry W.S. (2005). Mxi1 is induced by hypoxia in a HIF-1-dependent manner and protects cells from c-Myc-induced apoptosis. Cancer Biol. Ther..

[45] Wykoff C.C., Beasley N.J., Watson P.H., Turner K.J., Pastorek J., Sibtain A., Wilson G.D., Turley H., Talks K.L., Maxwell P.H. (2000). Hypoxia-inducible expression of tumor-associated carbonic anhydrases. Cancer Res..

[46] Muñoz-Nájar U.M., Neurath K.M., Vumbaca F., Claffey K.P. (2006). Hypoxia stimulates breast carcinoma cell invasion through MT1-MMP and MMP-2 activation. Oncogene.

[47] Choi J.Y., Jang Y.S., Min S.Y., Song J.Y. (2011). Overexpression of MMP-9 and HIF-1α in Breast Cancer Cells under Hypoxic Conditions. J. Breast Cancer.

[48] Cangul H. (2004). Hypoxia upregulates the expression of the NDRG1 gene leading to its overexpression in various human cancers. BMC Genet..

[49] Wang V., Davis D.A., Haque M., Huang L.E., Yarchoan R. (2005). Differential gene up-regulation by hypoxia-inducible factor-1alpha and hypoxia-inducible factor-2alpha in HEK293T cells. Cancer Res..

[50] Imai T., Horiuchi A., Wang C., Oka K., Ohira S., Nikaido T., Konishi I. (2003). Hypoxia attenuates the expression of E-cadherin via up-regulation of SNAIL in ovarian carcinoma cells. Am. J. Pathol..

[51] Liu K.-H., Tsai Y.-T., Chin S.-Y., Lee W.-R., Chen Y.-C., Shen S.-C. (2018). Hypoxia Stimulates the Epithelial-to-Mesenchymal Transition in Lung Cancer Cells Through Accumulation of Nuclear β-Catenin. Anticancer Res..

[52] Y W., L L., X S., W L., R M. (2021). Plakophilin-2 Promotes Lung Adenocarcinoma Development via Enhancing Focal Adhesion and Epithelial-Mesenchymal Transition. Cancer Manag. Res..

[53] Ym L., Yh W., Jt H., Yj L., Yl H., Gs L., Yl H., Jc W., Al Y. (2021). High B3GALT5 expression confers poor clinical outcome and contributes to tumor progression and metastasis in breast cancer. Breast Cancer Res..

[54] S L., P C., R E., J C. (2023). Mechanisms and roles of podosomes and invadopodia. Nat. Rev. Mol. Cell Biol..

[55] Y L., P L., Y L., W G. (2024). A novel gene-based model for prognosis prediction of head and neck squamous cell carcinoma. Heliyon.

[56] Brooks D.L.P., Schwab L.P., Krutilina R., Parke D.N., Sethuraman A., Hoogewijs D., Schörg A., Gotwald L., Fan M., Wenger R.H., Seagroves T.N. (2016). ITGA6 is directly regulated by hypoxia-inducible factors and enriches for cancer stem cell activity and invasion in metastatic breast cancer models. Mol. Cancer.

[57] A N., A O., Ky C., I L., Rc S., J P., S F., Sp W., C C. (2021). Palmitate-Induced IRE1-XBP1-ZEB Signaling Represses Desmoplakin Expression and Promotes Cancer Cell Migration. Mol. Cancer Res. : MCR.

[58] Wang V., Davis D.A., Yarchoan R. (2017). Identification of functional hypoxia inducible factor response elements in the human lysyl oxidase gene promoter. Biochem. Biophys. Res. Commun..

[59] Pez F., Dayan F., Durivault J., Kaniewski B., Aimond G., Le Provost G.S., Deux B., Clézardin P., Sommer P., Pouysségur J., Reynaud C. (2011). The HIF-1-inducible lysyl oxidase activates HIF-1 via the Akt pathway in a positive regulation loop and synergizes with HIF-1 in promoting tumor cell growth. Cancer Res..

[60] Zhang H.-J., Tao J., Sheng L., Hu X., Rong R.-M., Xu M., Zhu T.-Y. (2016). Twist2 promotes kidney cancer cell proliferation and invasion by regulating ITGA6 and CD44 expression in the ECM-receptor interaction pathway. OncoTargets Ther..

[61] Winkler J., Abisoye-Ogunniyan A., Metcalf K.J., Werb Z. (2020). Concepts of extracellular matrix remodelling in tumour progression and metastasis. Nat. Commun..

[62] Oppegard S.C., Blake A.J., Williams J.C., Eddington D.T. (2010). Precise control over the oxygen conditions within the Boyden chamber using a microfabricated insert. Lab Chip.

[63] Mosadegh B., Lockett M.R., Minn K.T., Simon K.A., Gilbert K., Hillier S., Newsome D., Li H., Hall A.B., Boucher D.M. (2015). A paper-based invasion assay: assessing chemotaxis of cancer cells in gradients of oxygen. Biomaterials.

[64] Chang C.-W., Cheng Y.-J., Tu M., Chen Y.-H., Peng C.-C., Liao W.-H., Tung Y.-C. (2014). A polydimethylsiloxane-polycarbonate hybrid microfluidic device capable of generating perpendicular chemical and oxygen gradients for cell culture studies. Lab Chip.

[65] Dimitriou N.M., Flores-Torres S., Kyriakidou M., Kinsella J.M., Mitsis G.D. (2024). Cancer cell sedimentation in 3D cultures reveals active migration regulated by self-generated gradients and adhesion sites. PLoS Comput. Biol..

[66] Li J., Kretzner L. (2003). The growth-inhibitory Ndrg1 gene is a Myc negative target in human neuroblastomas and other cell types with overexpressed N- or c-myc. Mol. Cell. Biochem..

[67] Schito L., Rey-Keim S. (2025). Transcriptional regulation of hypoxic cancer cell metabolism and artificial intelligence. Trends Cancer.

[68] Schito L., Semenza G.L. (2016). Hypoxia-Inducible Factors: Master Regulators of Cancer Progression. Trends Cancer.

[69] Schito L., Rey S. (2022). Hypoxia orchestrates the lymphovascular-immune ensemble in cancer. Trends Cancer.

[70] Han S.J., Kwon S., Kim K.S. (2021). Challenges of applying multicellular tumor spheroids in preclinical phase. Cancer Cell Int..

[71] Fares J., Fares M.Y., Khachfe H.H., Salhab H.A., Fares Y. (2020). Molecular principles of metastasis: a hallmark of cancer revisited. Signal Transduct. Target. Ther..

[72] Pfaffl M.W. (2001). A new mathematical model for relative quantification in real-time RT-PCR. Nucleic Acids Res..

[73] Ruijter J.M., Ramakers C., Hoogaars W.M.H., Karlen Y., Bakker O., van den Hoff M.J.B., Moorman A.F.M. (2009). Amplification efficiency: linking baseline and bias in the analysis of quantitative PCR data. Nucleic Acids Res..

[74] Vandesompele J., De Preter K., Pattyn F., Poppe B., Van Roy N., De Paepe A., Speleman F. (2002). Accurate normalization of real-time quantitative RT-PCR data by geometric averaging of multiple internal control genes. Genome Biol..

