# Supplementary material for: HyPIC-3D enables characterization of migratory cancer cell subpopulations in 3D hypoxic microenvironments
Source: Cell Rep Methods. 2026 May 11;6(6):101454. doi: 10.1016/j.crmeth.2026.101454 (PMC13282655; doi:10.1016/j.crmeth.2026.101454)
Supplement: Document S1. Figures S1–S5 and Tables S1–S3 [file mmc1.pdf]

Cell Reports Methods, Volume *6*

Supplemental information

HyPIC-3D enables characterization of migratory cancer cell subpopu-lations in 3D hypoxic microenvironments

Luana Schito and Sergio Rey-Keim

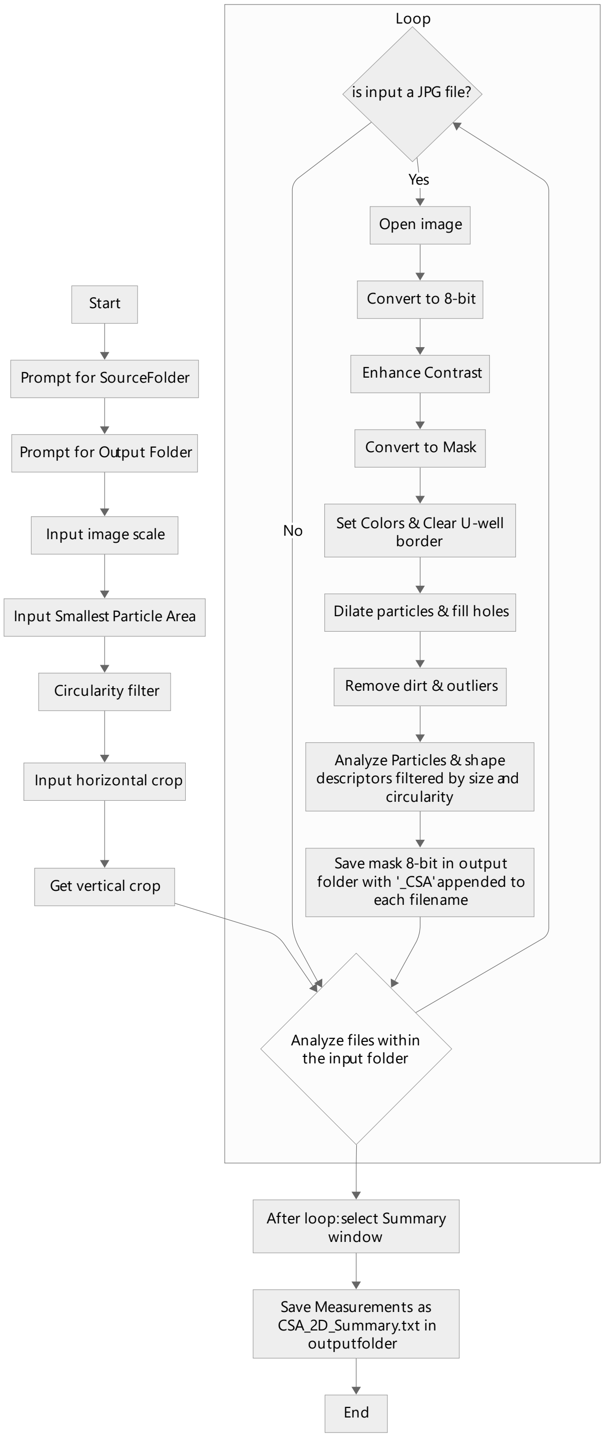

Figure S1. Flowchart of HyPIC-3D algorithm for spheroid image analysis at growth endpoint (day 4), related to Figure 1 and Figure 2.

Flowchart mapping the automated steps executed by HyPIC-3D code for extraction of morphometric data from spheroid brightfield images (script 01).

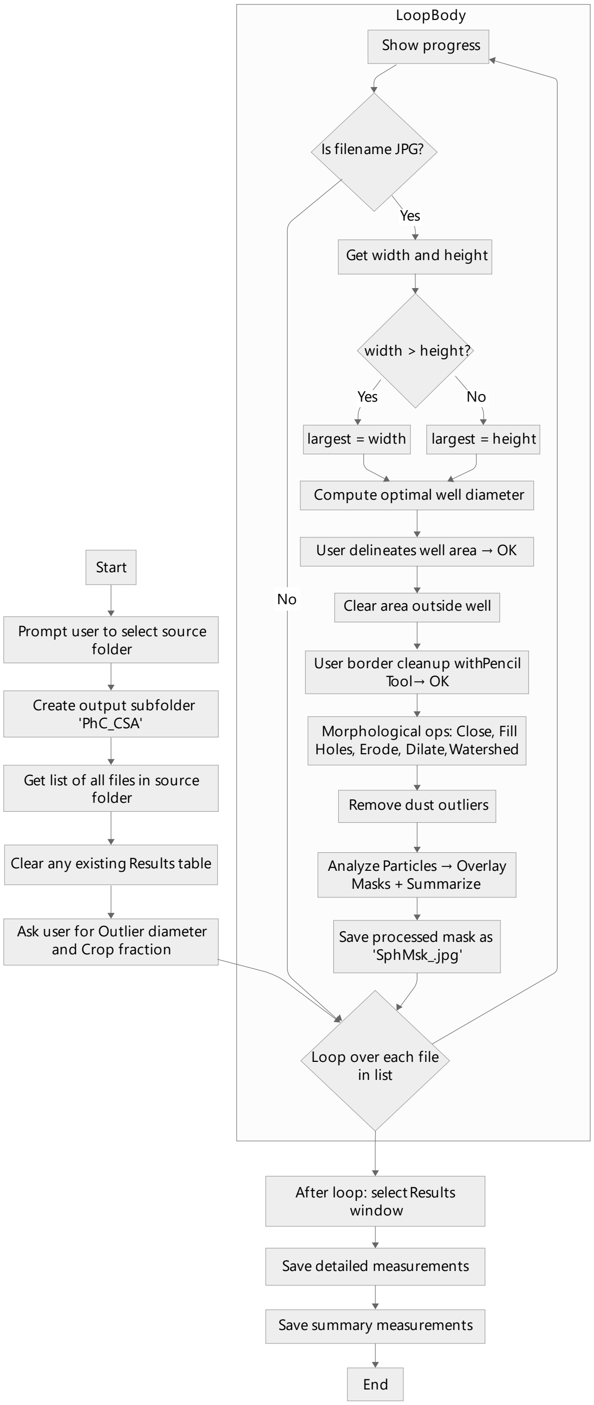

Figure S2. Flowchart of HyPIC-3D algorithm for image analysis of spheroids at migration endpoint, related to Figure 1 and Figure 2.

Flowchart mapping the semi-automated steps of HyPIC-3D code determining migration cross-sectional area (CSA) using brightfield images (script 02).

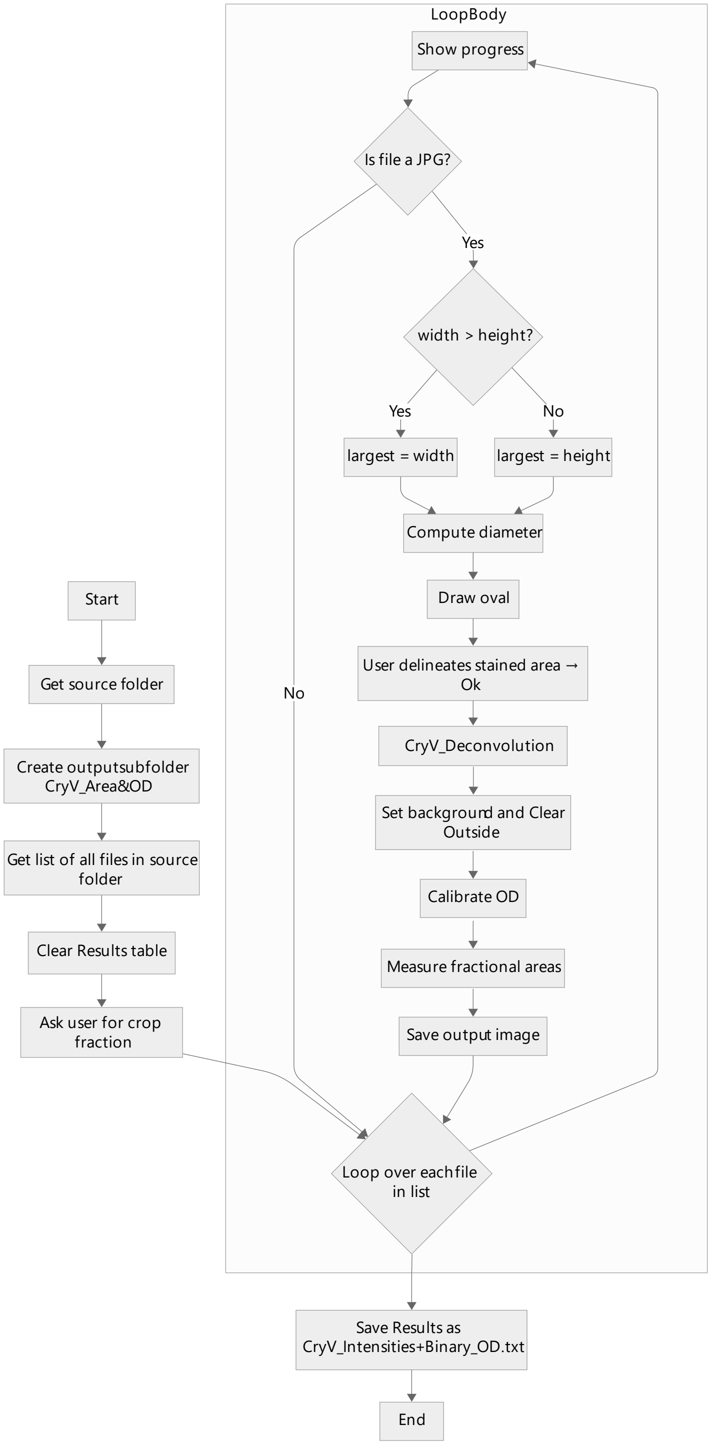

Figure S3. Flowchart of HyPIC-3D algorithm for image analysis of post-migrated, crystal violet-stained spheroids, related to Figure 1 and Figure 2.

Flowchart mapping the semi-automated steps of HyPIC-3D code for determining crystal violet intensities in migrated spheroid fractions (script 03).

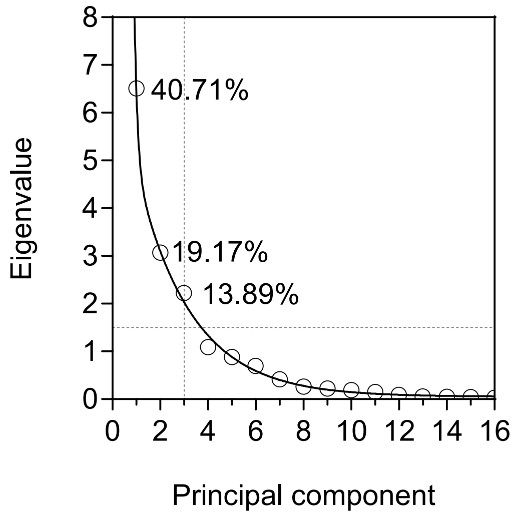

Figure S4. Principal component analysis (PCA) of gene expression data, related to Figure 3.

Scree plot illustrating the distribution of variance (eigenvalues) as a function of principal components.

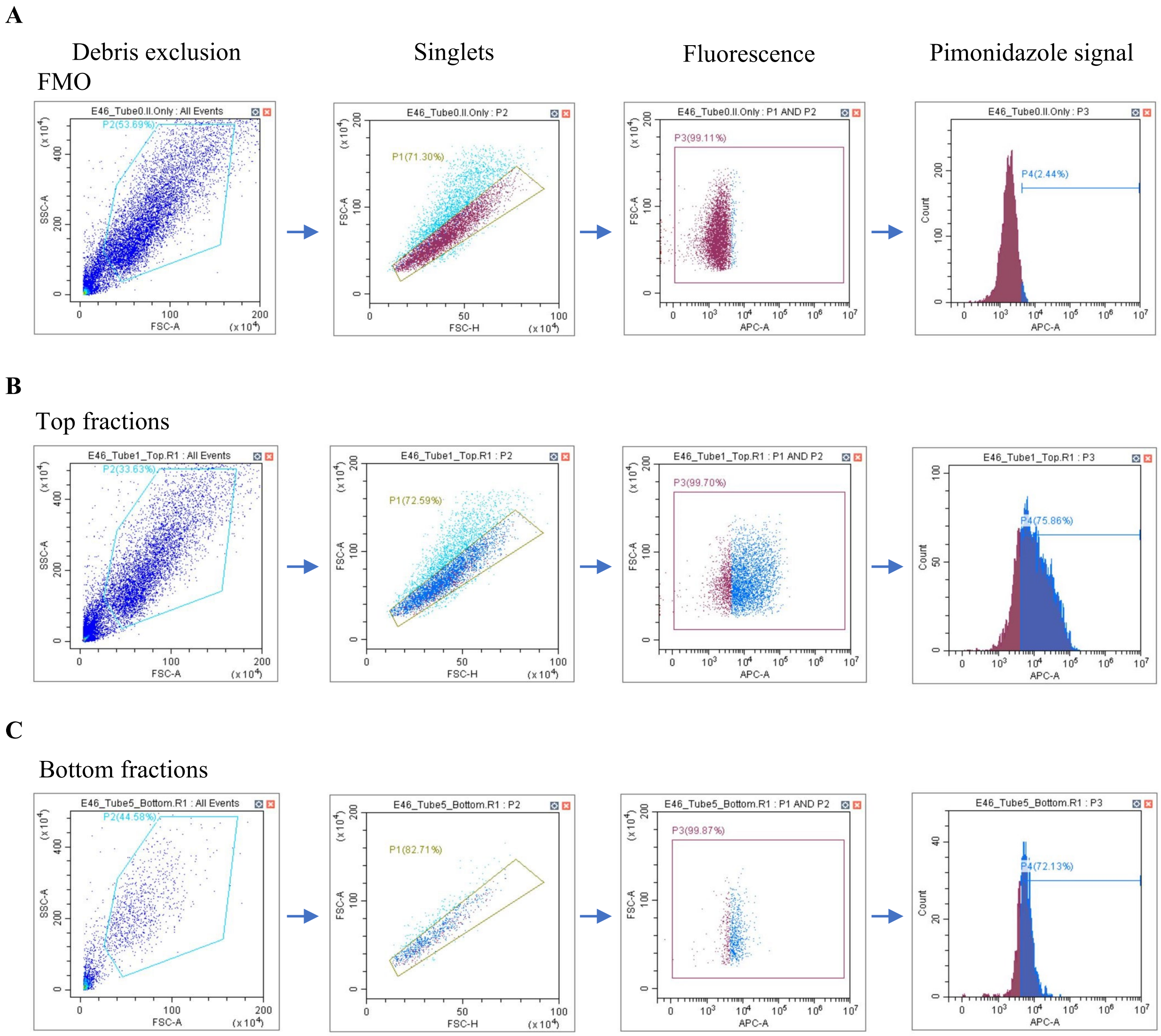

Figure S5. Flow cytometric gating strategy for detection of intracellular hypoxia in non-migrated and migrated spheroid fractions, related to Figure 4.

(**A**-**C**) Flow cytometric gating applied to either unstained cell fractions (**A**), stained non-migrated fractions (top fractions; **B**), or stained migrated fractions (bottom fractions, **C**). FMO, fluorescence minus-one (negative control).

Transcript	PC1	PC2	PC3	PC4	PC5	PC6	PC7	PC8
CA9	0.355	0.004	0.137	-0.008	0.024	-0.168	0.174	-0.401
DSP	0.343	-0.176	0.012	0.038	-0.066	-0.085	-0.322	-0.102
TJP3	0.332	0.194	-0.133	0.046	0.197	-0.004	-0.118	-0.422
MMP9	0.317	-0.028	0.060	-0.140	0.169	-0.518	-0.246	0.265
VIM	0.302	-0.104	0.279	-0.142	-0.299	-0.104	0.307	0.068
PKP2	0.292	-0.228	-0.102	-0.177	0.063	0.396	-0.093	0.068
NDRG1	0.284	-0.045	-0.323	-0.299	-0.245	0.109	0.193	0.018
ACTB	0.282	0.179	-0.304	-0.214	0.165	0.230	-0.075	0.124
MMP2	0.270	0.079	-0.099	0.499	0.229	0.041	0.383	0.587
ITGA6	0.234	0.405	-0.014	0.177	0.055	0.202	-0.260	0.028
LOX	0.224	-0.156	0.474	0.108	0.054	-0.197	0.028	0.036
CDH2	0.100	0.090	-0.289	0.402	-0.758	-0.159	-0.123	-0.005
B3GALT5	0.083	-0.365	0.186	0.513	0.073	0.407	-0.118	-0.237
MXI1	0.072	-0.492	0.022	-0.208	-0.179	0.243	0.032	0.181
CDH1	-0.006	-0.290	-0.471	0.145	0.237	-0.255	0.449	-0.309
PARD6B	-0.091	-0.414	-0.316	0.098	0.141	-0.260	-0.447	0.157

Table S1. Raw loadings of migration- and hypoxia-inducible- related transcripts using paired principal component analysis (PCA), related to Figure 3.

Individual loadings at PC1 to PC8 are shown for each quantified transcript. PC, principal component.

Rank	PC1	PC2	PC3
1	*CA9* [0.355]	*ITGA6* [0.405]	*LOX* [0.474]
2	*DSP* [0.343]	*TJP3* [0.194]	*VIM* [0.279]
3	*TJP3* [0.332]	*ACTB* [0.179]	*B3GALT5* [0.186]
4	*MMP9* [0.317]	*CDH2* [0.090]	*CA9* [0.137]
5	*VIM* [0.302]	*MMP2* [0.079]	*MMP9* [0.06]
6	*PKP2* [0.292]	*CA9* [0.004]	*MXI1* [0.022]
7	*NDRG1* [0.284]	*MMP9* [-0.028]	*DSP* [0.012]
8	*ACTB* [0.282]	*NDRG1* [-0.045]	*ITGA6* [-0.014]
9	*MMP2* [0.270]	*VIM* [-0.104]	*MMP2* [-0.099]
10	*ITGA6* [0.234]	*LOX* [-0.156]	*PKP2* [-0.102]
11	*LOX* [0.224]	*DSP* [-0.176]	*TJP3* [-0.133]
12	*CDH2* [0.100]	*PKP2* [-0.228]	*CDH2* [-0.289]
13	*B3GALT5* [0.083]	*CDH1* [-0.290]	*ACTB* [-0.304]
14	*MXI1* [0.072]	*B3GALT5* [-0.365]	*PARD6B* [-0.316]
15	*CDH1* [-0.006]	*PARD6B* [-0.414]	*NDRG1* [-0.323]
16	*PARD6B* [-0.091]	*MXI1* [-0.492]	*CDH1* [-0.471]

Table S2. Ranked loadings of migration- and hypoxia-inducible- related transcripts using paired principal component analysis (PCA), related to Figure 3.

PC1-3, principal components 1-3, transcripts are ordered in descending order based on their loadings (brackets) in PC1, PC2 or PC3 considering an absolute modulus larger than 0.3 as significant and highlighted in red (positive modulus) or blue (negative modulus).

Gene symbol	Forward (5' – 3')	Reverse (5' – 3')	Amplicon size (bp)
ACTB	GGGTCAGAAGGATTCCTATGTGGG	TGAAGGTCTCAAACATGATCTGGGT	242
CA9	AGAGGATCTACCTACTGTTGAGGC	GACTCTGGTCATCCCCTTCTTTG	89
B3GALT5	CTCTGGGAATGGTAAGAACAAGGA	GAATCCACATTCTGCACTTCTAGC	86
CDH1	TTCACCATTAACAGGAACACAGGA	GTATACGTAGGGAAACTCTCTCGG	77
CDH2	AGGAGAAGAAGACCAGGACTATGA	GTCTTTCATCCATTCGTCGGATTC	104
DSP	GCTTCAGACAAAATTGAGGCCTAT	AACATCAATGCACTTGGTGATCTG	84
ITGA6	ATACCAAACCAACACAGGTTCTCA	TCAGGGTAGGAATTTCGATCAAGG	96
LOX	TGGCTACCACAGGCGATTTG	GGGGTTTACACTGACCTTTAGGA	151
NDRG1	TCTTCAACTACGAGGACATGCAG	CCAATAATGCTTTTCAGCCCAAAC	181
MMP2	CTACGATGGAGGCGCTAATGG	TTCAGGTATTGCACTGCCAACT	169
MMP9	TCCAGTACCGAGAGAAAGCCTA	ACTGCAGGATGTCATAGGTCAC	117
MXI1	AACGAATACGAATGGACAGCATTG	AACATCCACTTCAATCTCCTCTCG	83
PARD6B	GCAGACATCCATGGAGACTTACTA	ACTGTAGTCTGCTTCTTCCTTCTTT	120
PKP2	AAGGAGACTACCCAAAAGCAAATG	CAGCAGAACTCATGTTTCTTAGGC	87
TJP3	GGTGATTGCAGAAAAAGACAAGCA	ATGGGGTAGTACTGCACATAGTTG	87
VIM	TGCAATCTTTCAGACAGGATGTTG	TTCCTCTTCGTGGAGTTTCTTCAA	116
B2M	GCAAGGACTGGTCTTTCTATCTCT	CTGCTTACATGTCTCGATCCCAC	135
RPL13A	TGAAGCCTACAAGAAAGTTTGCCT	GGTAGTGGATCTTGGCTTTCTCTT	120
TBP	AATCATGAGGATAAGAGAGCCACG	AGTCTGGACTGTTCTTCACTCTTG	96
YWHAZ	ACAAGCAGAGAGCAAAGTCTTCTA	TGACTGATCGACAATCCCTTTCTT	106

Table S3. List of RNA primers used in HyPIC-3D, RT-qPCR-based transcriptional analysis, related to STAR Methods.

ACTB, actin beta; *CA9*, carbonic anhydrase 9; *B3GALT5*, beta-1,3-galactosyltransferase 5; *CDH1*, cadherin 1; *CDH2*, cadherin 2; *DSP*, desmoplakin; *ITGA6*, integrin subunit alpha 6; *LOX*, lysyl oxidase; *NDRG1*, N-myc downstream regulated 1; *MMP2*, matrix metallopeptidase 2; *MMP9*, matrix metallopeptidase 9; *MXI1*, MAX interactor 1, dimerization protein; *PARD6B*, par-6 family cell polarity regulator beta; *PKP2*, plakophilin 2; *TJP3*, tight junction protein 3; *VIM*, vimentin; *B2M*, beta-2-microglobulin; *RPL13A*, ribosomal protein L13a; *TBP*, TATA-box binding protein; *YWHAZ*, tyrosine 3-monooxygenase/tryptophan 5-monooxygenase activation protein zeta.
